# Overcoming Therapeutic Resistance in Head and Neck Squamous Cell Carcinoma (HNSCC): The Role of Histone Methyltransferase and Demethylase Inhibitors

**DOI:** 10.3390/cancers18132170

**Published:** 2026-07-06

**Authors:** Kamila Adamczuk, Paulina Miziak, Grzegorz Adamczuk, Marzena Baran, Matthias Nees, Andrzej Stepulak

**Affiliations:** 1 Department of Biochemistry and Molecular Biology, Medical University of Lublin, 20-093 Lublin, Poland; paulina.miziak@umlub.edu.pl (P.M.); marzena.baran@umlub.edu.pl (M.B.); andrzej.stepulak@umlub.edu.pl (A.S.); 2 Department of Physiology and Toxicology, The John Paul II Catholic University of Lublin, 20-708 Lublin, Poland; grzegorz.adamczuk@kul.pl

**Keywords:** head and neck squamous cell carcinoma, HNSCC, histone lysine methyltransferase, histone lysine demethylase, KMT, KDM, histone methylation, epigenetic modifications, histone demethylase inhibitors, histone methyltransferase inhibitors, therapeutic resistance

## Abstract

Despite advances in multimodal treatment, head and neck squamous cell carcinoma (HNSCC) remains a major clinical problem owing to its high recurrence rate and frequent development of treatment resistance. Abnormal histone modifications, particularly lysine methylation regulated by methyltransferases (KMTs) and demethylases (KDMs), have emerged as key drivers of HNSCC initiation, progression, and cellular plasticity. This review aims to comprehensively evaluate the role of selected KMTs and KDMs in HNSCC biology, with a focus on their contribution to resistance to immunotherapy, radiotherapy, and cytotoxic chemotherapy. We summarize and critically analyze preclinical and clinical studies investigating histone methylation dynamics in HNSCC, with particular emphasis on enzymes such as KMT2C/D, EZH2, NSD1/NSD2, SMYD3, G9a/EHMT2, LSD1, KDM2A/B, KDM3, KDM4, KDM5, KDM6, KDM7, and KDM8. Attention is given particularly to pharmacological approaches targeting these proteins: we discuss small-molecule inhibitors of EZH2, LSD1, KDM4/5/6, and other KMT/KDMs that are currently in preclinical development or in early clinical trials, and we highlight completed and ongoing studies testing EZH1/2 inhibitors and epigenetic combinations in patients with recurrent and metastatic HNSCC. The deregulation of specific KMTs and KDMs reshapes histone methylation at key residues, thereby controlling cell cycle progression, epithelial–mesenchymal transition (EMT), stem cell phenotypes, DNA damage responses, and multiple interactions with the immune system in HNSCC. Targeting disrupted histone methylation pathways may partially reverse the epigenetic reprogramming of HNSCC cells and represents a promising strategy to improve treatment efficacy in patients with advanced disease. We also summarize the preclinical evidence and the currently limited clinical data on targeting histone methylation dynamics in HNSCC and discuss their therapeutic implications.

## Simple Summary

Head and neck squamous cell carcinoma (HNSCC) is one of the most common malignancies worldwide. Despite advances in surgery, radiotherapy, chemotherapy, and immunotherapy, many patients still experience disease recurrence and treatment resistance. A growing body of research indicates that this resistance is driven not only by permanent gene mutations but also by reversible epigenetic modifications of histones. In this review, we focus on histone-modifying proteins—methyltransferases and demethylases—which act as switches controlling growth, the ability of cancer cells to repair DNA damage, and evade the immune system. Discussing these mechanisms will help identify new therapeutic targets and rationally design combination therapies that could enhance the effectiveness of current head and neck squamous cell carcinoma treatments.

## 1. Introduction

**Figure 1:**
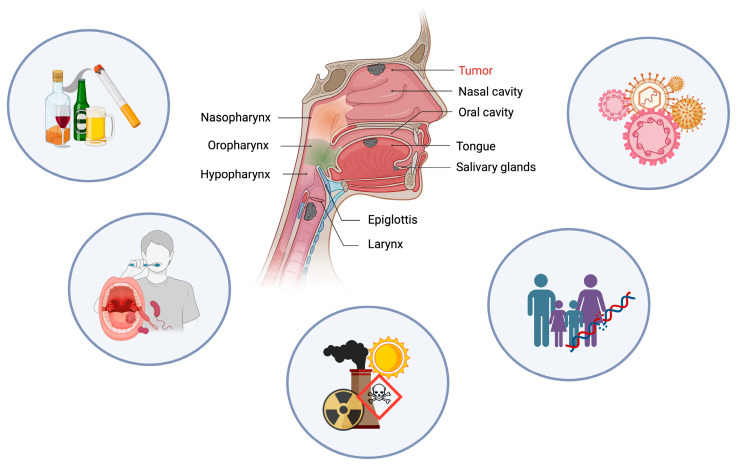
Anatomical subsites and main risk factors for HNSCC include tobacco and alcohol use, poor oral hygiene, oncogenic viruses, environmental exposures, and genetic predisposition.

Head and neck cancers comprise a heterogeneous group of malignancies arising in the oral cavity, pharynx, and larynx, most of which are squamous cell carcinomas (Figure 1). The most frequent tumor type represents squamous cell carcinoma (SCC) of the head and neck, or HNSCC. In this review, we focus on HNSCC of the oral cavity, oropharynx, hypopharynx, and larynx; nasopharyngeal carcinoma and salivary gland tumors are mentioned only where mechanistically relevant. According to GLOBOCAN 2022, head and neck cancers accounted for approximately 940,000 new cases and 480,000 deaths worldwide in 2022 [1].

The etiology of HNSCC is complex and multifactorial, involving both environmental and infectious factors. The most important risk factors are tobacco smoking and excessive alcohol abuse, which have a synergistic effect: joint exposure to these factors increases the risk of developing HNSCC [2]. Another important etiological factor is human papillomavirus (HPV) infection, particularly HPV-16, which plays a key role in the pathogenesis of oropharyngeal cancer. HPV-positive tumors have a different molecular profile and a better prognosis compared to HPV-negative tumors. HPV-encoded oncoproteins E6 and E7 inactivate key tumor suppressor proteins, including p53 and Rb, leading to uncontrolled cell division and neoplastic transformation [3]. The recent increase in HPV infections, especially in developed countries, has led to a significant increase in the number of oropharyngeal cancers not associated with the traditional risk factors such as tobacco and alcohol [4]. Another infectious factor, especially in nasopharyngeal cancer, is the Epstein–Barr virus (EBV), which can lead to neoplastic transformation through epigenetic mechanisms and stimulation of epithelial cell proliferation [5]. Occupational and environmental factors, such as exposure to wood dust, formaldehyde, heavy metals, and UV radiation, also influence the development of HNSCC. Genetic factors, although less well understood, may also predispose the oral mucosa to the development of HNSCC through inheritance of mutations in genes involved in DNA repair (Figure 1) [6,7,8].

The treatment of HNSCC depends on the stage of the disease, the location of the tumor, and the patient’s overall condition. The therapeutic approach differs significantly between the early and advanced stages of the disease and often requires a combination of treatments, such as surgery, radiotherapy, chemotherapy, and systemic immunotherapy. Early-stage HNSCC is typically treated with surgery, either alone or in combination with radiotherapy. In contrast, recurrent/metastatic head and neck squamous cell carcinoma (R/M HNSCC) is typically treated with cisplatin, 5-fluorouracil, and cetuximab as first-line chemotherapy (the EXTREME regimen) or with immune checkpoint inhibitors.

In contrast to conventional chemotherapy, targeted therapies focus on specific molecular targets involved in tumor pathogenesis and progression, allowing for more precise and often well-tolerated treatment. Currently, the only approved targeted therapy is epidermal growth factor receptor (EGFR) inhibitors. EGFR overexpression is observed in a large fraction of patients with HNSCC, but it varies significantly by tumor location, disease stage, and assessment method [9,10]. Cetuximab, a chimeric IgG1 monoclonal antibody directed against EGFR, blocks ligand binding and activation of signaling cascades, including by activation of protooncogenes like RAS/RAF/MEK/ERK and loss of tumor suppressors including PI3K/AKT/mTOR, which promote proliferation, angiogenesis, and survival of tumor cells. However, the use of cetuximab in combination with chemotherapy only slightly prolongs median overall survival (OS) from 7.4 to 10.1 months [11]. Another treatment option for HNSCC is immunotherapy with immune checkpoint inhibitors targeting the programmed death-1 (PD-1) receptor or the related PD-L1. These agents include pembrolizumab and nivolumab, which target the PD-1/PD-L1 pathway, thereby activating T lymphocytes and restoring the immune response against the tumor. In recurrent and metastatic HNSCC, pembrolizumab is now approved as a first-line treatment, either in combination with platinum and 5-fluorouracil for all patients or as monotherapy for tumors with PD-L1 expression (CPS ≥ 1), based on the successful KEYNOTE-048 trial and subsequent regulatory approvals [12]. Nivolumab is approved for patients with platinum-refractory recurrent or metastatic disease, as demonstrated in the CheckMate 141 study [13,14]. Other molecularly targeted drugs, such as HRAS inhibitors, VEGFR inhibitors, or newer EGFR/PI3K-pathway agents, largely remain experimental, investigational, or limited to highly selected molecular niches. None of them are currently part of standard treatment regimens. Nevertheless, many researchers believe that targeted therapies are the future of personalized medicine. In the future, this may include agents that interfere with epigenetic modifications and reprogram tumor cells, but the situation is anything but simple.

Despite advances in diagnostic imaging, surgical techniques, and the use of combination therapies, targeted therapies, and immunotherapy, the prognosis for patients with advanced HNSCC remains unfavorable [15]. Most patients develop resistance to treatment, even if they frequently have a positive initial response, leading to locally advanced or recurrent/metastatic disease [16]. The mechanisms of treatment resistance are multifactorial and involve both epigenetic and genetic factors, resulting in impaired DNA/RNA repair, increased cell proliferation, inhibition of apoptosis, and promotion of the cancer stem cell (CSC) phenotype [17]. In this context, epigenetic dysregulation is regarded as a particularly promising target [18]. At least eleven distinct post-translational modifications (PTMs), including methylation, acetylation, phosphorylation, and ubiquitination, occur on the amino acids of histones [19], most often lysine and arginine. Methylation, unlike other histone modifications such as acetylation and phosphorylation, was initially considered permanent. However, subsequent studies have shown that histone (lysine or “K”) methylation is also a highly dynamic process regulated by both histone methyltransferases (KMTs) and demethylases (KDMs) [20]. Recently, small molecules targeting these enzymes have entered clinical trials for cancer treatment (e.g., NCT06709885, NCT05546580). Several small-molecule KMT/KDM inhibitors are now in clinical development across multiple malignancies, including the EZH1/2 inhibitor Valemetostat (NCT05879484) in combination with pembrolizumab, and the KDM4 inhibitor Zavondemstat (TACH101, NCT05076552). Modulation of chromatin accessibility by targeting chromatin regulators, such as KMTs and KDMs, represents a promising strategy to overcome therapeutic resistance in HNSCC by reversing resistance-related cancer cell phenotypes and increasing the efficacy of existing treatments. Several KMT/KDM inhibitors have entered clinical development across malignancies (see Section 3.3); however, clinical evidence in HNSCC remains limited.

## 2. Molecular Basis of Histone Lysine Methylation and Demethylation

**Figure 2:**
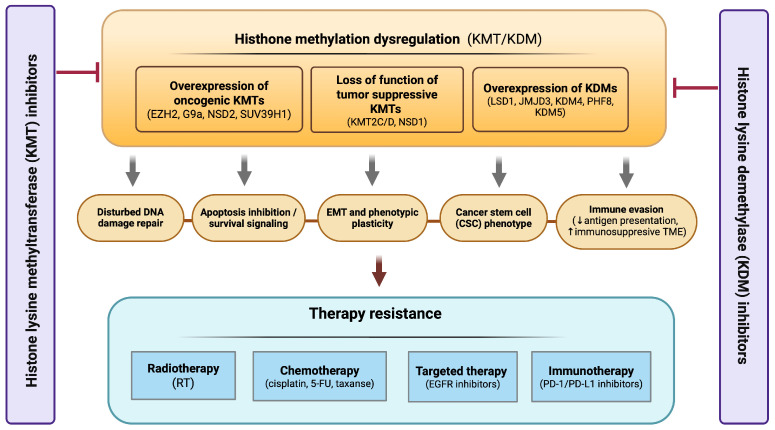
Histone lysine methylation dysregulation drives key resistance mechanisms (altered DNA repair, escape from apoptosis, EMT/CSC phenotype, immune evasion) and promotes resistance to radiotherapy, chemotherapy, EGFR-targeted therapy, and immunotherapy, all of which KMT/KDM inhibitors can counteract.

Histone methylation is a dynamic and reversible epigenetic mark controlled by histone lysine methyltransferases (KMTs) and demethylases (KDMs), which together modulate chromatin accessibility to transcription factors and regulate the expression of several genes, including oncogenes and tumor suppressor genes [21,22]. In the context of histone lysine methylation, these enzymes are often conceptualized as epigenetic “writers” (KMTs) and “erasers” (KDMs), which install and remove methyl groups on specific lysine residues, whereas a third class of chromatin factors acts as methylation “readers” that recognize distinct methyl-lysine states and recruit transcriptional co-regulators. Histone lysine methylation, best characterized on histone H3 (H3K4, H3K27, H3K36) and H4 (H4K20), can lead to both transcriptional activation and repression. Therefore, the biological effect of a given methyl mark is highly context-dependent and determined by the modified residue, the methylation state (me1/me2/me3), and the surrounding chromatin environment [23,24,25]. Methylation marks typically associated with active chromatin, such as H3K4me3 and H3K36me2/3, are frequently enriched at promoters and enhancers of genes involved in cell-cycle progression, DNA damage response, and survival pathways [26,27]. In turn, marks typically associated with transcriptional repression, such as H3K27me3 and H3K9me2/3, accumulate at loci encoding tumor suppressors, differentiation factors, and immune-response genes, thereby silencing their expression [28,29]. This combinatorial reorganization of activating and repressive histone marks contributes to the acquisition of characteristic features of HNSCC, including uncontrolled proliferation, epithelial-to-mesenchymal transition (EMT), maintenance of the CSC phenotype, and remodeling of the tumor microenvironment [21,22]. In HNSCC, recurrent alterations in genes encoding chromatin regulators, together with extensive reshaping of histone methylation patterns, reprogram transcriptional networks that sustain tumor growth and therapy resistance (Figure 2).

KMTs install methyl groups using S-adenosylmethionine (SAM) as a methyl donor. Most carry a conserved SET domain that confers substrate specificity. A notable exception is DOT1L, which methylates H3K79 via a structurally distinct, SET-independent mechanism [30,31,32,33]. These cofactor dependencies define the primary pharmacological entry point for KMT inhibition: SAM-competitive small molecules that occupy the SET domain’s substrate-binding channel.

KDMs remove methyl groups through one of two catalytic strategies. LSD1/KDM1A and LSD2/KDM1B use an FAD-dependent oxidase mechanism and are restricted to mono- and dimethylated substrates [34]. The larger Jumonji C (JmjC) family (KDM2-KDM8) employs an iron- and α-ketoglutarate-dependent hydroxylation mechanism and can also remove trimethyl marks [29,35,36,37,38,39]. These cofactor dependencies define the main pharmacological entry points: FAD-site inhibitors for LSD1/2 and iron- and 2-oxoglutarate-competitive inhibitors for JmjC domain-containing KDMs. In HNSCC, recurrent alterations in genes encoding these chromatin regulators, together with extensive reshaping of histone methylation patterns, reprogram transcriptional networks that sustain tumor growth and therapy resistance (Figure 2). How specific KMTs and KDMs drive these resistance programs—and how they can be pharmacologically targeted—is addressed in the following sections.

## 3. Shared KMT/KDM-Driven Resistance Programs in HNSCC

The following sections (Section 4 and Section 5) will establish that a large and growing set of histone lysine methyltransferases and demethylases is deregulated in HNSCC, and we will address the most relevant of these epigenetic writers and erasers in these paragraphs in a comprehensive and systematic fashion. Taken individually, each enzyme appears to contribute to its own characteristic biology. Viewed together, however, a more general picture emerges: this diverse enzymatic machinery converges on a relatively small number of recurrent resistance programs. The clinically most important question is not how many chromatin regulators (=candidate genes) are altered in HNSCC, but which downstream programs they jointly control, and whether those programs are pharmacologically vulnerable for interference; followed by the question in which patients (if any) interference with these processes is likely relevant. This section reframes the single enzyme-level evidence around those more central questions.

### 3.1. Many Enzymes, Few Programs: Convergence on Some Shared Resistance Phenotypes

Across the multiple enzymes and activities reviewed here, deregulated lysine methylation reshapes the same four resistance-associated programs (Figure 2). The logic is consistent: activating marks (H3K4me3, H3K36me2/3) are typically redistributed toward proliferation, survival, and DNA-repair loci. In contrast, the repressive histone marks (H3K27me3 and H3K9me2/3) typically accumulate at tumor suppressors and at genes that drive epithelial differentiation and immune response [26,27,28,29]. The same phenotypic output can, therefore, be reached by opposite enzymatic modifications: either by the gain of a repressive writer or by the loss of the corresponding eraser. This is the reason why a gene-by-gene analysis of histone methylases versus demethylases understates how tightly these enzymes are, in fact, functionally linked.

EMT and the cancer-stem-cell (CSC) phenotype represent the most relevant cell plasticity-related programs featured in this review, and there are a number of concrete examples pointing to the roles of histone demethylases and -transferases in lineage-specific differentiation versus increased tumor cell plasticity. LSD1 cooperates with SNAI1 to demethylate H3K4 at the E-cadherin promoter, thus driving EMT and sustaining cancer stemness [40,41,42]. In contrast, the methyltransferases G9a/EHMT2 reach the same endpoint through SNAI1-directed H3K9me2-mediated silencing of E-cadherin. Similarly, methyltransferase KMT2D supports stem-like traits via regulating Wnt/β-catenin expression and activity [43,44]. Finally, KDM6A, KDM7B, and KDM8 each independently promote EMT and invasion [45,46,47]. These findings show that mechanistically distinct epigenetic writers and erasers can still converge to increase EMT/CSC-related tumor cell plasticity via distinct histone marks and transcriptional routes. The observed phenotype, rather than any single enzyme activity, should therefore represent the most informative and relevant feature for biological and therapeutic interpretation.

Similar insights are observed in the regulation of DNA damage response and radioresistance. Several of the same enzymes can couple differential chromatin states to the DNA-damage response and are therefore relevant for radiotherapy outcome. For example, KDM6A/B activity opposes H3K27me3 accumulation, and inhibition of KDM6A/B with GSK-J1/J4 results in chromatin condensation and sensitizes HNSCC cells to irradiation [48]. Independently of this, loss of the histone methyltransferase G9a promotes TMEM27-dependent ferroptosis and markedly enhances radiosensitivity [49]. The most logical therapeutic implication in both examples is that different, and even directionally opposing chromatin perturbations can eventually converge on lowering the post-radiation survival threshold. But it is mechanistically distinct from, and occasionally opposite to, the EMT and increased tumor cell plasticity logic outlined above.

Similar opposing mechanisms can also converge at the regulation of apoptosis escape, redox adaptation, and chemoresistance. Resistance to platinum chemotherapy is often attributed to chromatin-regulated survival and altered redox pathways. The methyltransferase G9a transactivates the catalytic subunit of glutamate-cysteine ligase (GCLC), thereby increasing intracellular glutathione levels and promoting cisplatin detoxification. Its inhibition consequently resensitizes cisplatin-resistant cells. Using the opposite mechanism, the histone demethylase KDM4A can induce a proliferative, apoptosis-resistant state by activating LEF1-LATS2 expression and HIF1α/DDIT4/mTOR signaling [50,51]. Finally, KDM5B was shown to suppress Bcl-2-family-dependent positive regulation of apoptosis [52]. There is a shared theme of opposing mechanisms with similar effects. Histone methylases and demethylases do not simply promote growth, but they can cooperate tightly to buffer the cell against the specific stresses that are the immediate result of cytotoxic anti-cancer therapy.

Interaction and cooperation between histone demethylases and transferases also play a role in antigen presentation, interferon signaling, and immune exclusion. The most striking convergence between epigenetic writers (=methyltransferases) and erasers (=histone demethylases) has some clear implications for immunotherapy, namely, on the antigen-presentation and interferon axis. The well-investigated histone methyltransferase EZH2 represses MHC class I antigen-presentation genes, thereby lowering tumor visibility to cytotoxic lymphocytes [53]. Similarly, the histone methyltransferase SMYD3 represses tumor-intrinsic interferon responses. When SMYD3 is deleted, it derepresses IFN and antigen-processing machinery genes and can convert “cold” tumors (without prominent immune cell infiltration) into “hot” tumors with an intense presence of tumor-infiltrating lymphocytes (TILs). This also synergizes with anti-PD-1 therapy [54,55]. Although the mechanism acts in the opposite direction, inhibition of the lysine demethylase LSD1 also increases immunogenicity and potentiates PD-1 blockade [42]. Finally, loss of histone methyltransferase NSD1—frequently observed in HNSCC—leads to accumulation of H3K27me3 marks on promoters of cytokine genes like CXCL9/CXCL10, resulting in T-cell exclusion. This effect is directly reversed by inhibition of the histone demethylase KDM2A [56]. This expanded example shows that four enzymes acting on different histone methylation marks and opposing mechanisms (writers versus erasers) can still converge and cooperate or counteract within the same MHC-I/IFN/CXCL9-10 chemokine signaling mechanism; their activity can shift the balance between induction and blockade of immune infiltration. This example also demonstrates how mechanistically diverse KMT/KDM inhibitors can be but also shows that many of them may share a common rationale as potential sensitizers for immunotherapy.

The same enzyme can be oncogenic or tumor suppressive. This can heavily depend on the tumor location, epithelial versus mesenchymal differentiation state, and HPV status. For example, the histone methyltransferase KMT2D is often lost in laryngeal/hypopharyngeal tumors, but is more commonly overexpressed and pro-tumorigenic in oral squamous cell carcinomas OSCC/OPSCC [57,58,59]. Similarly, high activity of the histone demethylase KDM6A is associated with favorable outcomes in mixed cohorts dominated by radiotherapy response, but with adverse effects in oral-tongue SCC [45,48]. Finally, the demethylase KDM5D shows context-dependent behavior (see Section 5.5). In HPV-positive disease, the methyltransferases NSD1/NSD2/NSD3 act as suppressors or favorable markers [60,61,62,63]. Any synthesis of the functional roles of histone demethylases and methyltransferases that ignores this context dependence would be misleading. Some of these opposing therapeutic consequences are specifically addressed in more detail in Section 3.3 and Section 3.4.

A further implication of these findings is that these resistance programs are not inherent. They are not necessarily fixed properties of the untreated tumor. Instead, they may emerge, be replaced, or be unmasked during therapy. For example, inhibition of LSD1 suppresses stem-like properties while simultaneously inducing PD-L1. This replaces a CSC-associated tumor cell state with an adaptive immune-evasion state and provides the rationale for combining LSD1 inhibition with PD-1 blockade, as suggested in [42]. Platinum exposure can similarly enrich a KDM5D-high, drug-tolerant cell population whose collateral dependence on Aurora kinase B (AURKB) expression makes it vulnerable to AURKB inhibition and induction of mitotic catastrophe. However, this finding derives from a single HNSCC study and may be sex-restricted because KDM5D is Y-linked [64]. Models for radioresistant HNSCC also tend to enrich stem-like cell states that can be re-sensitized through KDM6A/B-directed intervention [48]. Collectively, epigenetic intervention may therefore redirect rather than simply eliminate resistance, and rational combinations should be designed against both the pre-existing mechanism and the adaptive state the intervention itself induces or selects.

### 3.2. Where Interference with Demethylases and Methyltransferases May Be Feasible—And Where It Likely Is Not

Two structural features make this demethylase versus transferase machinery more tractable than many oncology targets: (1) the marks are reversible, and (2) the enzymes typically carry defined cofactor pockets. These are S-adenosylmethionine or SAM for SET-domain KMTs, flavin-adenine dinucleotide or FAD for LSD1, and Fe (II)/2-oxoglutarate for the JmjC-family of KDMs [30,31,34]. Small molecules exploiting these pockets exist for most of the families covered here. In HNSCC models, they produce coherent, mechanistic effects KMT/KDM inhibitors targeting EZH2, LSD1, KDM4, KDM5, or KDM6 variously restrain cell proliferation and simultaneously induce apoptosis or senescence. They can also sensitize cells to radiotherapy, cisplatin, EGFR/PI3K blockade, or anti-PD-1 [42,48,65,66,67,68]—the list of examples is long but typically confined to pre-clinical models and experimentation. The recurrent finding that inhibition is most effective in combination rather than as a monotherapy is consistent with the convergence model: these enzymes set the threshold for resistance rather than autonomously driving growth or growth inhibition. Their functions are embedded in partially redundant and compensatory regulatory networks.

However, the obvious structural similarities and the parallels between enzyme activation and inhibition are not necessarily grounds for excessive optimism. Four caveats need to be considered that may temper this optimism. First, catalytic inhibition is not equivalent to successful protein inactivation: For example, SMYD3 is transcriptionally bifunctional, and just blocking catalysis alone may be insufficient. Instead, full removal of the protein from cells (e.g., via antisense oligonucleotides) may be required to show the desired effects [55,69]. Another example: KDM5B/JARID1B retains a demethylase-independent function that persists despite the action of its catalytic inhibitor, CPI-455 [70].

Second, multiple parallel on-target effects can be counterproductive: LSD1 inhibition successfully restrains CSC properties, but unfortunately, it simultaneously raises expression of PD-L1. This facilitates immune escape unless the anti-LSD1 strategy is combined with PD-1 blockade [42].

Third, much preclinical HNSCC data rests entirely on pan-KDM tool compounds (e.g., JIB-04) or on effects observed with non-selective agents (e.g., tranylcypromine, which also inhibits monoamine oxidases). These are pleiotropic, overlapping, and confounding effects that cannot be cleanly or clearly attributed to a single enzyme [51,71]. In other cases, where more selective probes or compounds have been used (ML324 for KDM4; CPI-455 for KDM5), the conclusions are simpler and easier to interpret.

Fourth, toxicity can constrain or remove a therapeutic option without invalidating the target class entirely. For example, the EZH2 inhibitor Tazemetostat was withdrawn globally in 2026 after a delayed safety concern about hematological second primary malignancies, reported in a confirmatory trial (by the FDA in 18 of 318 treated patients versus none in the control arm; SYMPHONY-1 trial). This demonstrates that epigenetic cell toxicity is not hypothetical, but a realistic risk factor. Nevertheless, it does not establish that any single molecular, genetic, or epigenetic mechanism was responsible for this [72], nor does it identify a specific candidate [72].

### 3.3. Mechanistic Rationale: Preclinical Efficacy vs. Clinical Actionability

An honest approach must distinguish three important issues that are easily mixed up: (1) mechanistic rationale, (2) preclinical efficacy, and (3) the true clinical actionability. First, the rationale is often strong and convergent, as argued above—but that is not sufficient nor is it decisive. Secondly, preclinical efficacy shown in HNSCC model systems is broad and may, at first, look convincing, but it rests overwhelmingly on established cell lines and animal xenografts. Thirdly, the demonstrated true clinical benefit in HNSCC is essentially absent at present. There is very little convincing evidence for successful interference with advanced HNSCC and benefits for patients. The single published HNSCC trial of a histone-methylation-targeting agent combined Tazemetostat with Pembrolizumab in recurrent/metastatic disease. This trial produced no objective responses (5/12 stable disease), enrolled an unselected, heavily pretreated population without biomarker stratification, and did not proceed to phase II [72]. The only remaining nominally active HNSCC program, Valemetostat plus Pembrolizumab (PANTHERAS, NCT05879484), is currently listed as withdrawn before efficacy data matured. As of this writing, no active small-molecule KMT/KDM program in HNSCC can be considered clinically established.

The negative result from the Tazemetostat trial is at least consistent with important limitations of trial design and patient selection: there was no requirement for evaluating the target expression, no readout related to measuring H3K27me3 marks or epigenetic regulation of target gene expression, no stratification of HPV and NSD1 mutation status, and the trial included a likely too advanced, highly refractory patient cohort with a probability of enrichment for the relevant biological vulnerability [72]. The lesson these data support is narrow and restricted, but still important: unselected, single-agent-style deployment of epigenetic inhibitors in late-line HNSCC is unlikely to succeed and should not be pursued in the future. Whether biomarker-directed trials based on drug combinations can succeed remains an open question. However, this may be the most urgent and most likely proposition the field should test.

The strength of experimental evidence differs markedly between these regulators. The NSD1/H3K36 axis is possibly the most independently reproduced finding. It defines a recurrent HPV-negative subset across separate molecular cohorts. Furthermore, it has been linked, by independent data from different groups, to DNA hypomethylation and an immune-cold microenvironment [73]. Another study linked this NSD1/H3K36 axis to enhanced cisplatin sensitivity and improved patient outcomes [74].

### 3.4. Toward Personalized Epigenetic Medicine in HNSCC

The heterogeneity (of patients, tumors, responses, etc.) documented throughout this review is typically presented as an obstacle. But it can also be seen as the precondition for a workable future strategy. Because KMT/KDM dependencies are specific to subtypes, tumor location, HPV status, genetic predisposition, and mutation spectrum, a single “one-size-fits-all” approach is unlikely to succeed without molecular selection. But at the same time, this exquisite specificity is exactly what makes biomarker-driven selection of patients and patient cohorts more plausible. The convergence model described above confirms this as a potentially working concept: if mechanistically very distinct or even opposing enzymes still share downstream programs, then the actionable target is the biological program, mechanism or pathway and its biomarker(s) or gene signatures as indicators for patient selection and stratification. Patient selection based solely on the expression and activity of a single enzyme is unlikely to be sufficient or informative; the relevant chromatin state and downstream resistance program should also be investigated and established.

HNSCC already provides at least one working example of this logic. NSD1 loss (by loss-of-function mutations) depletes intergenic H3K36me2 marks and triggers compensatory H3K27me3 accumulation. For this reason, NSD1-deficient tumors are predicted to be selectively sensitive to PRC2/EZH2 inhibition [75,76]. A second example is the immune-axis node elaborated in Section 3.1: Tumors with high expression and activity of the methyltransferases SMYD3 or EZH2 show low expression of the chemokines CXCL9/10 and suppressed antigen presentation. These tumors define a candidate patient cohort in which adding a KMT/KDM inhibitor, in combination with immune checkpoint blockade, has a realistic and coherent mechanistic rationale [42,54,56]. Future HNSCC trials should exploit this by: (i) establishing HPV/p16 status and alterations of chromatin-regulators for every patient enrolled in such studies; (ii) the trials require confirmed target expression or activity rather than enrolling the patients agnostically; (iii) successful trials will need to establish mandatory analysis of baseline and before, during and after treatment biopsies in which the relevant histone-mark readout (H3K27me3 for EZH2, H3K9me2 for G9a, H3K4me2 for LSD1) is firmly investigated. This is needed to confirm target engagement; (iv) pairing the anti-epigenetic agent with a rationally chosen clinical standard-of-care drug. Or therapy—this can be radiotherapy, platinum drugs (cisplatin and carboplatin), or EGFR/PI3K blockade (e.g., Cetuximab). The most promising rationale would be to combine such drugs with immune checkpoint inhibition; and (v) successful, sensitive trials should avoid heavily pretreated, advanced, cachexic, and epigenetically very heterogeneous populations.

The NSD1/H3K36 axis also illustrates why a single genotype alone may be insufficient for patient selection. Experimental HNSCC models deficient in H3K36 methylation can be separated into functionally distinct downstream chromatin states: those with pronounced H3K27me3 accumulation show impaired homologous recombination DNA repair, resulting in sensitivity to PARP1/2 inhibition. In contrast, models with steady H3K27me3 methylation marks lack this vulnerability unless H3K27me3 is pharmacologically increased, for example by inhibition of KDM6A/B [77]. Apparently, similar upstream alterations may produce different drug responses. These depend on the resulting landscape of histone marks and DNA repair competence observed in the cells. As a result, future biomarker strategies should combine genotyping of NSD1 or H3K36-pathway status with direct measurements of H3K36me2 and H3K27me3 histone methylation marks, DNA repair activity, HPV status, anatomical subsite, and hot/cold immune state. Emerging chromatin-regulator signatures support the benefit of this composite approach, but these studies remain retrospective and require prospective validation [78].

In short, histone-methylation-directed therapy in HNSCC is best understood not as a set of competing single agents but as a future strategy that is supported by the choice of the right, most informative biomarkers, the most promising drug combinations, and the most sensitive patient pre-screening and stratification. No such adequately designed clinical tests have been conducted yet. The mechanistic case for targeting histone methylation/demethylation may be strong, but the clinical case is still essentially unproven. The major gap that has yet to be bridged is answered as much by sensitive, informed trial design and patient selection as by the inherently complex yet very interesting biology.

Among the KMTs reviewed here, EZH2 has the most extensive clinical track record in HNSCC and therefore receives the most detailed treatment in this section. This reflects clinical maturity, not biological primacy. The evidence base for SMYD3, G9a/EHMT2, and NSD1 in the biology of acquired therapy resistance is mechanistically equally compelling, and LSD1, KDM4, and KDM6 carry comparably strong preclinical rationales. Where clinical data are sparse, the introduction of key players and the discussions below aim to explicitly distinguish the mechanistic rationale from the demonstrated preclinical efficacy and likely clinical actionability.

## 4. Targeting Histone Lysine Methyltransferases (KMTs) in HNSCC Therapy

### 4.1. KMT2D

**Figure 3:**
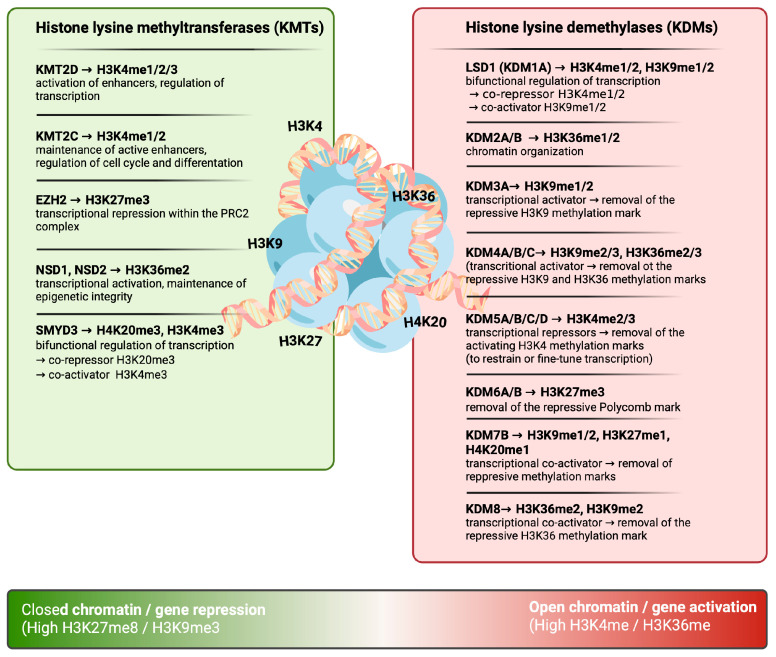
Schematic overview of KMTs and KDMs regulating key lysine residues (H3K4, H3K9, H3K27, H3K36, and H4K20), illustrating how their opposing activities shape closed, repressive chromatin versus open, transcriptionally active chromatin states.

KMT2D is the most frequently mutated epigenetic modifier gene in HNSCC, and its alteration profile includes, among others, frameshift, truncating, and missense mutations, suggesting substantial diversity of mutational events within this gene (Table 1) [79]. KMT2D exhibits a highly context-dependent dual role in HNSCC: in laryngeal and hypopharyngeal tumors, it is frequently affected by loss-of-function mutations, whereas, at the protein level, it is often overexpressed in OSCC and OPSCC, where it promotes progression and stemness. Both observations indicate that KMT2D function probably depends strongly on cellular and genetic context, HPV status, and other cooperating factors. KMT2D methylates H3K4 (mono-, di-, and trimethylation) (Figure 3), thereby activating enhancers and promoting cell-type-specific transcription, which in turn influences proliferation, differentiation, and cell-cycle homeostasis [80]. In HNSCC, KMT2D mutations disrupt these processes; however, high KMT2D expression levels support the development of a second primary tumor and cancer stem-like traits via the Wnt/β-catenin pathway (through cooperation with MEF2A and CTNNB1) [43,44]. High KMT2D expression in HNSCC, especially in oral squamous cell carcinoma (OSCC), correlates with tumor progression, metastasis, and poorer long-term prognosis; it is observed in tumor cells, fibroblasts, and infiltrating lymphocytes [57,58]. In HPV-positive oropharyngeal squamous cell carcinoma (OPSCC), high KMT2D expression is associated with improved survival, whereas overall KMT2D exhibits oncogenic activity by promoting invasion and maintaining stemness [59].

Beyond stemness, retained or high KMT2D activity has been linked to immune dysfunction and metastasis: KMT2D-dependent enhancer regulation supports CCL2 expression, tumor-associated macrophage recruitment, T-cell exhaustion, and lymph node metastasis. Loss of CCL2 partially reproduces this phenotype [81]. Whether KMT2D is a tumor suppressor or a tumor promoter/proto-oncogene largely depends on whether the tumor carries loss-of-function mutations (or deletion) of KMT2D or retains a KMT2D-dependent transcriptional program. This directly connects KMT2D activity and loss to the immune-exclusion program discussed in Section 3.1.

**Table 1:** Overview of key KMTs implicated in HNSCC.

Clinical/Therapeutic Significance	Biological Consequences	Changes in HNSCC	Main Epigenetic Function	Main Substrate	Enzyme
Molecular stratification marker; potential prognostic and predictive factor for immunotherapy response [57,58,59]	Deregulated cell-cycle control, impaired differentiation, and maintenance of cancer stemness promote tumor progression and intratumor heterogeneity.	Frequent loss-of-function mutations in laryngeal/hypopharyngeal subtypes; protein overexpression reported in OSCC and OPSCC associated with tumor progression, metastasis, and shorter overall survival	Activation of enhancers and regulation of cell-type-specific transcription	H3K4me1/2/3	KMT2D (MLL2)
Important component of patient stratification; may influence tumor immunogenicity and response to immune checkpoint inhibitors (ICIs) [71,82]	Reduced expression of antiproliferative genes, increased expression of pro-invasive programs, and increased genomic instability.	Frameshift, nonsense, and missense mutations leading to loss of function frequently co-occur with KMT2D alterations	Maintenance of active enhancers and control of cell-cycle and differentiation gene expression	H3K4me1/2	KMT2C (MLL3)
Unfavorable prognostic marker; validated therapeutic target (EZH2 inhibitors and combinations with chemotherapy, radiotherapy, and ICIs) [53,65,72,83,84,85,86,87]	Increased proliferation, EMT, metastatic potential, and hypoxia tolerance; reduced MHC-I expression and antigen presentation leading to immune escape	Overexpression in HNSCC tissue versus normal mucosa; correlated with advanced stage, lymph node metastases, and worse overall survival	Transcriptional repression within the PRC2 complex	H3K27me3	EZH2 (KMT6)
Potential targets for NSD inhibitors and components of epigenomic signatures predicting tumor aggressiveness [60]	Increased proliferation, invasion, and resistance to DNA damage and therapeutic stress	Overexpression in many cancers; in HNSCC: high expression in HPV-negative tumors, reduced NSD1/NSD2 expression in the HPV-positive subtype	Transcriptional activation and regulation of cell-cycle progression and survival pathways	H3K36me2	NSD1/NSD2
Promising therapeutic target; preclinical data demonstrates efficacy of anti-SMYD3 antisense oligonucleotides in combination with anti-PD-1 therapy [54,55,69,88]	Enhanced proliferation, EMT, and invasion; establishment of an immunosuppressive tumor microenvironment (reduced CD8+ T-cell infiltration, decreased chemokine expression, poor PD-1 response)	Overexpression in ~80% HPV-negative HNSCC; associated with aggressive phenotype and worse overall and progression-free survival	Bifunctional regulation of transcription as a co-repressor (H4K20me3) and a co-activator (H3K4me3)	H4K20me3; H3K4me3; non-histone substrates	SMYD3
Pro-oncogenic enzyme and potential therapeutic target; genetic or pharmacologic inhibition reduces EMT/CSC features, induces apoptosis, and resensitizes cisplatin-resistant HNSCC cells to chemotherapy [89,90,91]	Promotes EMT and acquisition of cancer stem cell-like traits through interaction with SNAIL; enhances migration, invasion and lymph-node metastasis; upregulates GCLC and glutathione synthesis, supporting cisplatin detoxification and chemoresistance	Overexpression in HNSCC; particularly high in tumors with EMT-like and CSC-like features; elevated expression and activity in cisplatin-resistant HNSCC cell lines	Deposition of repressive H3K9me1/2 at euchromatic loci, cooperation with GLP to establish facultative heterochromatin	H3K9me1/2	G9a (EHMT2)

### 4.2. KMT2C (MLL3)

Exome sequencing in cohorts of patients with laryngeal and hypopharyngeal cancer showed that KMT2C is among the most frequently mutated epigenetic genes in these HNSCCs. KMT2C has a mutation frequency of 3.5–8% in HNSCC and often co-occurs with KMT2D mutations. The mutations include both missense (49%) and nonsense (32%) variants, suggesting heterogeneous mechanisms of loss of function [92]. A pan-cancer meta-analysis showed that mutations in the KMT2 family, including KMT2C, co-occur with distinct tumor microenvironment signatures ranging from immunogenic to immunosuppressive, which may partly explain the heterogeneous responses to immunotherapy in HNSCC. Taken together, these data suggest that KMT2C, like KMT2D, is a vital component of the molecular stratification of HNSCC patients for prognostic and potentially predictive purposes [74].

KMT2C, functionally similar to KMT2D, catalyzes H3K4me1/2 at enhancers and is crucial for regulating the expression of numerous genes that control the cell cycle, epithelial differentiation, and genome stability [93]. In HNSCC, loss of KMT2C function is associated with reduced expression of cell-cycle-inhibitory gene signatures and increased expression of pro-proliferative and pro-invasive genes, thereby promoting tumor progression and heterogeneity [94]. Available evidence also suggests that KMT2C loss may exacerbate genomic instability, increase the mutational burden, and potentially affect tumor immunogenicity and the response to immune checkpoint inhibitors (ICIs) [82].

### 4.3. EZH2 (Enhancer of Zeste Homolog 2, KMT6)

EZH2, the catalytic subunit of the PRC2 complex responsible for H3K27 trimethylation (H3K27me3), is one of the best-characterized oncogenic KMTs in HNSCC [95]. Immunohistochemical studies in HNSCC patient populations have shown a higher proportion of EZH2-positive cells in tumor tissue than in adjacent mucosa, which correlates with increased proliferative potential of the cancer [96]. In large clinical cohorts, high EZH2 expression has been associated with advanced clinical stage, more frequent lymph node metastases, and shorter OS, supporting its adverse prognostic significance [83]. A study of young HNSCC patients found EZH2 expression in this group significantly lower than in the general patient population, suggesting that EZH2’s role as a prognostic marker may depend on age and environmental exposures [84,85]. These findings indicate the need for more precise patient stratification when designing clinical studies with EZH2 inhibitors. Patients could be selected based on high-level EZH2 expression in tumor tissues, and/or NSD1-deficient epigenetic signatures, and further stratified according to age, HPV status, and prior exposure to chemotherapy and immunotherapy. Including mandatory baseline expression analysis of biopsies before and during treatment, ideally combined with an assessment of EZH2/H3K27me3 status of key EZH2-responsive genes, along with immune profiling, would also help identify patient subgroups that may derive the greatest benefit from EZH2 inhibition. For such studies, the biomarker-driven combination strategies should also be refined and better understood: what genes are predominantly affected by altered H3K27me3 methylation that results in changes in expression and activity, and what are the expected phenotypes? Mechanistically, EZH2 promotes HNSCC cell survival and progression through multifaceted, parallel, and connected epigenetic mechanisms. EZH2 overexpression has been shown to increase cell proliferation, hypoxia tolerance, and metastatic capacity. In part, this is due to the repression of tumor suppressor genes and the modulation of interactions between cancer cells and the extracellular matrix (ECM) [86]. Furthermore, EZH2 reduces the immunogenicity of cancer cells by suppressing the expression of genes involved in MHC class I antigen presentation, thereby decreasing the tumor’s “visibility” to cytotoxic lymphocytes and potentially contributing to resistance to immunotherapy [53].

Recent studies highlight that EZH2 dysregulation is likely linked to multiple oncogenic phenotypes. This ranges from loss of cell-cycle control to remodeling of the tumor microenvironment (TME). It is widely agreed that dysregulated EZH2 likely carries prognostic significance across many cancers, including HNSCC [87]. However, exactly how this can be exploited to target tumor cells for therapy remains poorly understood. The published data provide strong justification for developing EZH2 inhibitors and for their rational combination with immunotherapy and chemoradiotherapy in patients with high EZH2 expression [65]. Clinical translation of EZH2 inhibition in cancers, and specifically in HNSCC, is ongoing, but the success of these clinical trials to date has been limited (Table 2). The only completed HNSCC trial to date (Tazemetostat plus Pembrolizumab in recurrent/metastatic disease) produced no objective responses (5/12 stable disease). This trial was conducted in an unselected, heavily pretreated patient cohort, and the compound was subsequently withdrawn globally in 2026 after secondary hematologic malignancies were reported in the confirmatory SYMPHONY-1 lymphoma trial. These design limitations and the withdrawal are further analyzed and discussed in Section 3.2 and Section 3.3 and summarized in Table 2.

### 4.4. NSD1/NSD2

Histone methyltransferases NSD1 and NSD2 belong to the SET-domain KMT family and are key regulators of H3K36 methylation, which in turn affects chromatin organization, gene expression, and antitumor response. NSD1 is the primary H3K36-specific methyltransferase in HNSCC, whereas NSD2 modulates the H3K36me2 landscape at selected genes and regulatory elements [100]. The biochemical basis of NSD substrate specificity and its role in oncogenic programming is reviewed elsewhere [101,102].

NSD1 is a histone methyltransferase primarily responsible for the dimethylation of lysine 36 on histone H3 (H3K36me2) in intergenic regions [103]. Recurrent NSD1 mutations are identified in a subset of HNSCC and define a distinct molecular subgroup of these tumors. (Epi)genomic studies and CRISPR-based genome editing have shown that NSD1 loss leads to the depletion of intergenic H3K36me2 domains. This is accompanied by reduced DNA methylation and a compensatory increase in H3K27me3, resulting in a global reorganization of the chromatin landscape. It was also observed that the affected regions are enriched for cis-regulatory elements and that loss of H3K27ac, together with changes in DNA methylation, results in reduced expression of their target genes, including pathways involved in immune responses, EMT, and RAS signaling [75]. These pleiotropic changes may facilitate HNSCC development by simultaneously affecting antitumor immunity, cellular plasticity, and pro-growth signaling [76]. Since NSD1 is often lost in HNSCC, it does not represent a therapeutic target. However, from a translational perspective, NSD1-deficient tumors may represent a particularly well-justified subgroup of patients that could qualify for EZH2-directed therapy. This is because the loss of H3K36me2 (which is the primary target for NSD1) results in the accumulation of compensatory H3K27me3 marks. Epigenomic reprogramming driven by NSD1 loss may create specific therapeutic vulnerabilities, for example, to PRC2/EZH2 inhibitors or agents that modulate EMT- and RAS-signaling-related pathways. Although direct clinical data in HNSCC remain limited, analogies from other cancers suggest that NSD1 mutations may modulate responses to systemic therapy and immunotherapy, warranting further prospective studies [75].

NSD2 also catalyzes H3K36me2, but, in contrast to NSD1, its activity is highly enriched within gene bodies and at active enhancers, where it promotes transcription of genes involved in regulating cancer cell proliferation, survival, and tumor cell plasticity. For example, NSD2 was shown to increase H3K36me2 and decrease H3K27me3 at the *TWIST1* promoter, thereby inducing EMT, migration, and invasion. Its silencing partially reverses this phenotype (an increase in E-cadherin and a decrease in N-cadherin and vimentin) [76,104]. In HNSCC, recent analyses of large patient cohorts have shown a clear, context-dependent effect by HPV status: in HPV-positive HNSCC, low NSD2 expression (similar to NSD1 and NSD3) is not associated with changes in lymphocytic infiltration but correlates with significantly worse overall survival. In contrast, this relationship is not observed in HPV-negative tumors. This suggests that in HPV-positive HNSCC, NSD2 may act as a marker of favorable prognosis and thus represents a potential biomarker for patient stratification [60]. Regarding therapeutic relevance and clinical evidence level, NSD1 and NSD2 are perhaps best understood as recurrent genetic alterations that define patient subgroups rather than as direct pharmacological targets. NSD1 loss is among the strongest HNSCC-specific biomarkers for patient stratification: it identifies a specific chromatin state characterized by low H3K36me2, and H3K27me3 methylation marks, but “cold” immune status—with low levels of TIL invasion. All these parameters predict cisplatin sensitivity but poor checkpoint blockade response. This combination creates a synthetic vulnerability that may be exploited by inhibition of either EZH2 or KDM2A. Direct NSD1/2 inhibitors remain in the early stages of development and lack HNSCC-specific clinical data—the evidence level is low to moderate. The most actionable NSD1/2-related strategy at present is to use NSD1 mutation status or H3K36me2/H3K27me3 status as mandatory stratification criteria for future EZH2 inhibitor trials, or as a marker for immune-combination trials, rather than targeting NSD enzymes directly.

Several independent lines of evidence reinforce this stratification logic. CRISPR-mediated NSD1 disruption in HNSCC cells leads to CpG island hypomethylation and a 40–50% reduction in cisplatin IC50, with NSD1-mutant patients showing improved outcomes [74]. Furthermore, NSD1/NSD2 loss-of-function mutations define a subset of patients with favorable-prognosis, specifically of laryngeal carcinomas, that is unfortunately not reproduced across other tumor sites [105]. Conversely, in HPV-negative models with NSD1-wild-type functions, the retained NSD1 functions support Akt-mTORC1 signaling, autophagy, and cell growth [106]. This is mediated in part by H3K36me2-dependent regulation of PIP4K2B, an effect that is also restricted to tumor subsites [107]. NSD1-deficient and NSD1-retained or dependent HNSCC therefore represent distinct biological states that require differential or even opposing therapeutic strategies. Future stratification should therefore distinguish loss-of-function cases from disease with retained activity, rather than grouping all tumors with altered NSD1 functions together.

### 4.5. SMYD3

SMYD3 (SET and MYND domain-containing 3) is a histone methyltransferase with dual epigenetic functions, and its role in the pathogenesis of HPV-negative head and neck squamous cell carcinoma (HNSCC) has recently been clarified by a series of translational studies [54]. As a methyltransferase, SMYD3 catalyzes trimethylation of histone H3 lysine 4 (H3K4me3), a classic mark of active chromatin. However, SMYD3 also influences deposition of H4K20me3, a marker of transcriptional repression. Therefore, SMYD3 displays transcriptional bifunctionality, acting as both an activator (via H3K4me3) and a repressor (via H4K20me3). This implies that complete removal of the protein may be required to achieve full pharmacologic blockade [69]. This leads to the next complication: the SMYD3 protein functions both in the nucleus, where it directly binds gene promoters and recruits RNA polymerase II, and in the cytoplasm, where it methylates non-histone substrates. SMYD3 overexpression occurs in approximately 80% of HPV-negative HNSCC and correlates with an aggressive clinical phenotype, including higher tumor grade and a propensity for metastasis. Prognostically, high SMYD3 expression is an independent marker of poor outcome for OS and progression-free survival (PFS), and it also predicts a weak response to neoadjuvant pembrolizumab immunotherapy. In multivariable gene-expression analyses of patients treated with anti-PD-1 therapy, higher baseline SMYD3 levels were associated with a lower rate of pathological response (*p* = 0.02) and reduced CD8+ T-cell infiltration in biopsy specimens [54]. In HNSCC, SMYD3 depletion reduces the invasive potential of cancer cells, and genomic mapping identified direct SMYD3 targets at the promoters of genes that regulate cell migration [69].

At the functional level, SMYD3 regulates the cell cycle by transactivating pro-proliferative genes, particularly cyclins and inhibitors of cyclin-dependent kinase (CDK). In HPV-negative HNSCC cell lines, SMYD3 knockdown induces S-phase arrest, reduces expression of cell-cycle genes, and significantly limits colony-forming capacity ex vivo [69].

SMYD3 expression is significantly elevated in OSCC tissues compared to normal tissues. Its over-expression correlates with advanced histologic stage, TP53 mutations, smoking and alcohol abuse history, and the classical HNSCC subtypes. The underlying drivers for SMYD3 over-expression include copy-number amplification (CNV) and promoter DNA hypomethylation of SMYD3, as confirmed in TCGA datasets and clinical specimens using qRT-PCR, Western blotting, and IHC. High SMYD3 expression predicts worse OS and PFS [88].

In vivo, SMYD3 depletion using antisense oligonucleotides (ASOs) in the syngeneic MOC1 model (*C57BL/6 mice*) was able to convert the tumor immune phenotype from “cold” to “hot”: the highest CD8+ T-cell infiltration was observed at 12.5 mg/kg, accompanied by increased MHC-I expression on tumor cells combined with higher PD-L1 expression. The combination of Smyd3 ASO (12.5 mg/kg) with anti-PD-1 produced particularly compelling results: 50% of mice achieved complete cures, 25% showed marked regression, while 25% displayed therapeutic escape. Immunological changes included a substantial increase in CD8+ T cells, CD4+ T cells, and macrophages within the tumor microenvironment (TME) [55]. In HPV-negative HNSCC xenografts with SMYD3 knockout (KO), tumor growth was significantly slower compared with controls. Transcriptomic analysis of SMYD3 KO cells showed upregulation of 40 of 97 IFN-α-inducible genes and increased expression of 22 of 88 genes involved in the antigen-processing and presentation (APM) machinery. This was observed even in the absence of exogenous IFN-β, suggesting a LINE-independent mechanism of de-repression of the interferon-response. Clinical data support translation of these findings, although none have yet included ASOs targeting SMYD3. In TCGA cohorts, high SMYD3 mRNA levels correlate negatively with CD8A, the chemokines CXCL9/10/11, and interferon-stimulated gene (ISG) signatures. In a study testing the neoadjuvant use of pembrolizumab, higher SMYD3 expression predicted a poor pathological response. Multigene signatures that include SMYD3 have been predictive of OS, PFS, and response to immunotherapy [54].

### 4.6. G9a/EHMT2

G9a (EHMT2) and its paralogue GLP (EHMT1) are SET-domain lysine methyltransferases that catalyze mono- and dimethylation of H3K9 (H3K9me1/me2), thereby establishing a repressive chromatin state at euchromatic loci [108]. Although not part of the KMT2 family, G9a is directly relevant to HNSCC resistance. G9a is closely linked to the regulation of EMT, cancer stemness, the DNA damage response, and immune evasion. Mechanistically, G9a acts as an SNAI1-interacting partner that mediates H3K9me2-dependent silencing of E-cadherin, thereby driving EMT and maintaining cancer stem cell-like characteristics in HNSCC. As expected, the genetic depletion of G9a reverses EMT, suppresses tumor-spheroid or organoid formation, and reduces CSC marker expression [89]. In parallel, pharmacological or genetic inhibition of G9a induces DUSP4-dependent autophagic cell death in HNSCC cells without activating classical apoptotic pathways, accompanied by corresponding growth inhibition in orthotopic models [90].

G9a also contributes directly or indirectly to therapy resistance. High G9a expression is significantly associated with poor chemotherapeutic response and shorter disease-free survival in HNSCC patients. Furthermore, G9a expression and activity are often elevated in cisplatin-resistant HNSCC cell lines. Functionally, G9a transactivates the glutamate-cysteine ligase catalytic subunit (GCLC) and upregulates intracellular glutathione synthesis, thereby promoting cisplatin detoxification. Genetic or pharmacological inhibition of G9a again sensitizes resistant cells to cisplatin [91]. These data identify G9a/EHMT2 as a potential epigenetic driver of tumor cell plasticity in HNSCC, including EMT and partial EMT, CSC maintenance, cisplatin resistance, and radioresistance. Selective G9a/GLP inhibitors have been developed, including experimental compounds/inhibitors such as UNC0638, BIX-01294, BRD4770, and EZM8266. Some of these drugs have shown encouraging preclinical activity, especially in combinations with cisplatin, radiotherapy, and immune checkpoint blockade. These preliminary results warrant dedicated evaluation in HNSCC models and may encourage the first clinical trials in humans soon.

## 5. Histone Lysine Demethylases (KDMs) as Therapeutic Targets in HNSCC

### 5.1. LSD1/LSD2

LSD1/KDM1A (BHC110/AOF2/KDM1A) is a flavin-dependent epigenetic eraser that demethylates H3K4me1/2 or H3K9me1/. LSD1 can act as either a co-repressor or co-activator depending on its associated complex and transcriptional context. In its repressive mode, LSD1 operates within the CoREST/HDAC1/2-containing complex, where it couples H3K4 demethylation to histone deacetylation and gene silencing [109,110,111,112]. Through its dual function as a co-repressor and a co-activator, LSD1 is a central regulator of tumor cell proliferation and stem cell maintenance; it promotes enhanced tumor plasticity via increased EMT and CSC [113,114,115,116].

A comprehensive immunohistochemical analysis of a large, clinically well-characterized cohort of 339 patients with HNSCC demonstrated that elevated LSD1 expression is significantly associated with advanced tumor stage and reduced PFS, particularly in hypopharyngeal cancers (Table 3 and Table 4). Furthermore, elevated LSD1 levels in patient samples were significantly associated with increased SNAI1 expression, a strong promoter of EMT. The interaction between LSD1 and SNAI1 is crucial for LSD1 recruitment to the E-cadherin promoter and subsequent silencing of its expression via H3K4 demethylation. This, in turn, results in the loss of epithelial features, induction of EMT, and increased migratory and invasive capacity of cancer cells. Both proteins were overexpressed in laryngopharyngeal squamous cell carcinomas, a finding that correlates with an unfavorable prognosis and suggests their potential as new therapeutic targets in this tumor group [40,41]. Other studies demonstrate that LSD1 is overexpressed in tongue squamous cell carcinoma, the most common site of OSCC, highlighting its importance in the carcinogenesis of this group of head and neck cancers.

Moreover, LSD1 depletion increased the sensitivity of the OSCC cells studied to 5-FU, suggesting the potential use of this strategy in combination therapy to enhance the efficacy of standard chemotherapy [66,117]. The first studies focusing on pharmacological inhibition of LSD1 in OSCC were published by Alsaqer et al., 2017 [118]. The studies used three different LSD1 inhibitors: tranylcypromine (TCP), a non-selective LSD1 inhibitor that also inhibits monoamine oxidases A and B, and two selective LSD1 inhibitors: GSK-LSD1 and LSD1-C76. All three compounds suppressed tumor cell proliferation, with GSK-LSD1 demonstrating the greatest efficacy. These studies enabled a more comprehensive characterization of LSD1 function, revealing that its expression correlates with disease progression and tumor stage and that it acts as a key regulator of metastasis in OSCC. Pharmacological inhibition of LSD1 further decreased activity of the EGF signaling pathway and reduced the expression of several key genes associated with increased tumor cell plasticity and EMT, including CTGF/CCN2, MMP13, LOXL4, and vimentin. Simultaneously, LSD1 inhibition increased E-cadherin expression in patient-derived tonsillar OSCC xenografts (PDX), clearly demonstrating and validating that LSD1 regulates genes involved in EMT and remodeling of the tumor microenvironment [118]. The pharmacological effect of the LSD1-inhibitor TCP consisted primarily of inhibiting tumor growth and proliferation; the therapy did not induce tumor regression due to low efficacy at low doses. Therefore, a promising strategy may be to use LSD1 inhibitors in combination therapy, for example, pairing drugs like TCP with another anticancer compound to increase treatment efficacy, lowering drug concentrations, while (hopefully) simultaneously reducing side effects. Synergistic antitumor effects in HNSCC were also observed with TCP and GSK-J1, which simultaneously inhibit LSD1 and JMJD3. This strategy leads to impaired proliferation, induction of apoptosis and senescence in tumor cells, and inhibits tumor growth in preclinical models.

Among the indirect modulators, the natural compound melatonin has been reported to lower LSD1 expression and inhibit OSCC proliferation in PDX models with low toxicity [119]. Other studies have shown that overexpression of LSD1 in HNSCC cells promotes the acquisition of CSC characteristics by regulating Bmi-1, thereby increasing tumorigenesis and immune resistance. LSD1 inhibition limited CSC properties and tumor proliferation while simultaneously increasing PD-L1 levels, facilitating immune escape in immunocompetent models. Preclinical studies have shown that combining an LSD1 inhibitor with PD-1 blockade yields a significantly better therapeutic effect than monotherapy, accompanied by a decrease in the percentage of Ki-67-positive cells. These data highlight the complex role of LSD1 in HNSCC progression and suggest the potential of therapeutic strategies involving the combination of an LSD1 inhibitor with anti-PD-1/PD-L1 immunotherapy [42].

Overall, LSD1/KDM1A carries one of the strongest biological rationales in this field, supported by a patient IHC cohort with 339 patients, further validated by multiple HNSCC/OSCC functional models and PDX data. Its clinical translation depends on two conditions already detailed in Section 3.2, Section 3.3 and Section 3.4: (1) validation with selective inhibitors (e.g., GSK-LSD1, Pulrodemstat, and Iadademstat) rather than the non-selective inhibitor tranylcypromine, and (2) the mandatory combination with PD-1/PD-L1 blockade to offset the induction of PD-L1. No trial with an HNSCC-specific LSD1 inhibitor has yet been registered.

LSD2/KDM1B, the second FAD-dependent H3K4me1/2 demethylase, has been documented to play roles in other cancers but remains underexplored in HNSCC [120,121,122,123,124,125].

**Table 3:** Overview of key KDMs implicated in HNSCC.

Enzyme	Clinical/Therapeutic Significance	Biological Consequences	Changes in HNSCC	Main Epigenetic Function	Main Substrate
LSD1 (KDM1A)	Marker of unfavorable prognosis; target for LSD1 inhibitors (e.g., tranylcypromine, GSK-LSD1, LSD1-C76); promising combinations with chemotherapy and immune checkpoint inhibitors [40,41,42,43,66,118,119]	Silencing of tumor suppressor genes; promotion of EMT (via cooperation with SNAI1); increased proliferation and invasion; maintenance of cancer stem-cell populations	Overexpression at multiple HNSCC sites (tongue, pharynx, larynx) correlates with higher malignancy grade and worse progression-free survival.	Co-repressor (H3K4) and co-activator (H3K9); regulation of EMT, CSC and cell-cycle genes	H3K4me1/2; H3K9me1/2
KDM2A; KDM2B	KDM2A: restoration of CXCL9/10 expression and lymphocytic infiltration in NSD1-deficient HNSCC; KDM2B: potential target in HPV-dependent cancers (needs validation) [56,126,127]	Regulation of Ink4a/Arf axis, PRC1/2 and EZH2; impact on proliferation, senescence and tumor immune signatures	KDM2B overexpression is described in selected HNSCC cohorts; KDM2A is implicated in OSCC progression and immunomodulation in NSD1-deficient tumors	Chromatin organization, transcriptional control, and regulation of senescence	H3K36me1/2
KDM3A (JMJD1A)	Potential therapeutic target in selected HNSCC subtypes; requires further molecular stratification and functional validation [67,128]	Upregulation of ADM and other pro-oncogenic genes, increased proliferation and metastatic potential, in NPC, regulated by miR-155	KDM3A overexpression is reported in subsets of HNSCC, associated with lymph-node metastasis and poorer prognosis; context-dependent data in nasopharyngeal carcinoma (NPC)	Removal of repressive H3K9 marks and activation of pro-proliferative and pro-angiogenic genes	H3K9me1/2
KDM4A; KDM4B; KDM4C	Attractive target for pan-KDM inhibitors (JIB-04) and selective KDM4 inhibitors (ML324, myricetin, BPRKD022S0/22S0) [50,51,68,129]	Enhanced proliferation and invasion with reduced apoptosis; contribution to metabolic adaptation and stress resistance	KDM4A overexpression in OSCC and NPC; in HNSCC linked to activation of LEF1-LATS2 and HIF1α/DDIT4/mTOR signaling axes	Chromatin relaxation and regulation of cell-cycle, proliferation, metabolism, and stress-response genes	H3K9me2/3; H3K36me2/3
KDM5A; KDM5B; KDM5C; KDM5D	In OSCC, KDM5B is a preclinical target for the KDM5 inhibitor CPI-455; clinical exploitation requires molecular stratification of patients [52,70,130,131]	Modulation of E2F-dependent cell-cycle genes and pro-survival/stress-response pathways; KDM5D exerts context-dependent effects (tumor-suppressive in some settings, resistance-promoting in others)	KDM5B overexpression in HNSCC is associated with tumor progression, lymph-node metastasis, recurrence, and shorter overall and recurrence-free survival	Removal of “activation” marks from promoters and activation of pro-proliferative transcriptional programs (KDM5D shows context-dependent roles, including resistance-promoting functions)	H3K4me2/3
KDM6A (UTX); KDM6B (JMJD3)	KDM6 proteins are promising targets for JMJD3/UTX inhibitors (GSK-J1/J4) in combination regimens; further HNSCC-specific studies are needed [45,48,132]	Activation of EMT, inflammatory and survival programs; involvement in radio- and chemoresistance, particularly under hypoxic stress	KDM6A and KDM6B overexpression in OTSCC and ESCC; correlation with lymph-node metastasis and reduced overall and recurrence-free survival	Removal of repressive PRC2 marks; activation of EMT, differentiation, and pro-inflammatory genes	H3K27me3
KDM7B (PHF8)	Potential unfavorable prognostic marker and emerging therapeutic target [46,133]	Enhanced EMT, invasion, and proliferation; mechanistically linked to TGF-β and Wnt/β-catenin pathway activation	Overexpression in laryngeal HNSCC is associated with advanced tumor stage, recurrence, and shorter overall and recurrence-free survival	Regulation of cell cycle, EMT, metabolism, and stress responses	H3K9me1/2; H3K27me1; H4K20me1
KDM8	Unfavorable prognostic and metastasis marker; attractive therapeutic target (including the MTA1-KDM8 axis); KDM8 inhibition by silibinin proposed for OSCC/HNSCC therapy [47,134]	Increased migration, invasion, and survival; EMT reversal and p53-dependent apoptosis after KDM8 silencing; reduced nuclear NF-κB activity	Overexpression in OSCC correlates with larger tumor size, lymph-node metastasis, and shorter overall survival	Regulation of cell cycle, EMT, and p53/NF-κB signaling	H3K36me2; H3K9me2

**Table 4:** Evidence and priority ranking of KMT/KDM regulators in HNSCC therapeutic resistance. Tiers reflect combined strength, reproducibility, and clinical maturity of HNSCC-specific evidence, not biological importance. Tier 1 = best-supported, biomarker-trackable axes; for EZH2, Tier 1 denotes relative maturity (withdrawn drug, negative trial), meaning these enzymes/inhibitors candidates are best-positioned for biomarker-defined re-testing, not necessarily for validated benefit.

Regulator (Writer/Eraser)	Resistance Program(s)	Link	Reproducibility (Verified, Sets 1–4)	Highest Evidence Level	Tier	Key Refs
EZH2 (writer, H3K27me3)	Immune exclusion (MHC-I repression); proliferation, EMT, hypoxia	Strong	Multiple HNSCC cohorts + HNSCC-specific inhibitor efficacy (GSK-343 all lines; DZNeP HPV- only); cross-tumour prognostic	Early-phase HNSCC trial (negative; tazemetostat withdrawn 2026)	1	[53,65,72,83,84,85,86,87,97,98,135]
LSD1/KDM1A (eraser, H3K4/H3K9me1/2)	EMT/CSC; immune (PD-L1 rebound, single study); chemo-sensitisation	Strong	339-pt IHC + multiple HNSCC/OSCC models + PDX + selective inhibitor (Pulrodemstat); mitigates TCP-only caveat	Preclinical: selective KDM1A inhibitor in trials (other cancers)	1	[40,41,42,43,72,97,118,119]
NSD1 loss (↓H3K36me2 → ↑H3K27me3)	Immune exclusion (CXCL9/10); platinum sensitivity; synthetic vulnerability (EZH2/KDM2A)	Strong	Strongest in review: ≥6 independent groups, multiple cohorts, cross-tumor (lung SCC) and cross-subsite (larynx)	Biomarker-supported; CRISPR-causal; direct targeting preclinical	1	[56,60,73,74,75,76,103,105,107]
G9a/EHMT2 (writer, H3K9me1/2)	EMT/CSC; cisplatin resistance (GCLC/glutathione); radioresistance	Strong	Multiple HNSCC studies (Snail/EMT, GCLC/cisplatin, autophagy)	Preclinical only	2	[49,89,90,91]
SMYD3 (writer, H3K4me3/H4K20me3)	Immune exclusion (IFN/APM repression); EMT, proliferation	Strong	Oncogenic role multi-group; but immune/IFN mechanism concentrated in one group	Preclinical (ASO; catalytic inhibition alone is insufficient)	2	[54,55,69,88]
KDM4A/KDM4C (eraser, H3K9/H3K36)	Proliferation/apoptosis escape; EGFR-PI3K resistance; KDM4C-GATA1-FECH	Moderate	Repeated across OSCC + NPC, but many rests on pan-KDM JIB-04; selective ML324/myricetin firmer	Early clinical (Zavondemstat basket; no HNSCC subgroup)	2	[50,51,68,129]
KDM6A/KDM6B (eraser, H3K27me3)	Radioresistance (most consistent); EMT, plasticity	Moderate	Multiple but subsite-conflicting; radio-sensitization confirmed; dual LSD1/JMJD3 synergy	Preclinical (GSK-J1/J4 tool compounds)	2	[45,48,132]
KDM2A (eraser, H3K36me1/2)	Immune exclusion—restores CXCL9/10 in NSD1-deficient tumors	Moderate	Single key study; same group as NSD1 immune-cold work; not yet independently reproduced	Preclinical	2	[56,126,127]
KMT2D (writer, H3K4me1/2/3)	Stemness (Wnt/β-catenin, MEF2A); immune/metastasis; most-mutated modifier	Moderate	Multi-group, peer-reviewed; supersedes Callahan preprint	Biomarker/stratification (mutated or lost)	3	[43,44,57,58,59,79,81]
KMT2C (writer, H3K4me1/2)	Genomic instability; immunogenicity/ICI response	Preliminary	Cross-cohort sequencing + pan-cancer meta + KMT2 systematic review	Biomarker/stratification	3	[71,79,82,92,93]
KDM5B (eraser, H3K4me2/3)	Proliferation (E2F, Bcl-2); EMT (Wnt/β-catenin); CSC; recurrence	Moderate	Multiple independent HNSCC studies (Wnt/EMT; laryngeal cohort)	Preclinical (CPI-455; demethylase-independent function persists)	3	[52,70,130,131]
KDM5D (eraser; context-dependent)	Marks platinum-TOLERANT cells; vulnerability to mitotic catastrophe	Preliminary	NEW (was a gap): single HNSCC study with resistance-relevant functional data	Preclinical	3	[61,62,63,64]
KDM3A (eraser, H3K9me1/2)	Proliferation/metastasis (ADM); context-dependent (NPC miR-155)	Preliminary	Single HNSCC + single NPC report (opposite directions)	Preclinical	3	[67,128]
KDM7B/PHF8 (eraser)	EMT, invasion, proliferation (TGF-β, Wnt)	Preliminary	Single HNSCC (laryngeal) prognostic study confirmed	Preclinical/prognostic	3	[46,133]
KDM8/JMJD5	EMT; p53/NF-κB; metastasis	Preliminary	Two OSCC studies (incl. MTA1-KDM8/silibinin)	Preclinical	3	[47,134]

Research gaps/not ranked: KDM3B/C, KDM4B/D, KDM5A/C, KDM6C, KDM7A/C, LSD2/KDM1B (unexplored in HNSCC); plus recent single-group/in vitro candidates SUV420H1/KMT5B and SETDB1.

### 5.2. KDM2A/KDM2B

KDM2A and KDM2B are among the earliest described JmjC-demethylases and catalyze the demethylation of H3K36me1/2, thereby influencing chromatin organization and gene transcription. KDM2B was identified as a regulator of cellular senescence, whose overexpression promotes proliferation and protects against senescence. This mechanism involves repression of the Ink4a/Arf genes through demethylation of H3K36me2/H3K4me3 and increased recruitment of the PRC1/2 complex, leading to inhibition of expression of p15Ink4b, p16Ink4a, and p19Arf. Additionally, KDM2B suppresses the expression of miRNAs (let-7b, miR-101) that regulate EZH2, thereby secondarily enhancing its pro-proliferative effects [136,137].

Overexpression of KDM2B is observed in HPV16-positive cervical cancer cell lines and in a subset of laryngeal cancers, where it promotes tumor proliferation and progression. The high-risk HPV16 oncoproteins E6 and, to a lesser extent, E7 increases KDM2B expression in keratinocytes by overexpressing c-MYC and downregulating miR-146a-5p, which normally inhibits KDM2B. These results indicate that KDM2B is a key mediator of HPV-dependent transformation and may represent a potential therapeutic target in these tumors [126]. Recent studies have demonstrated that the circular RNA circFOXO3 plays a significant role in OSCC progression by modulating the miR-214/KDM2A axis. RNA analyses revealed that circFOXO3 is highly overexpressed in OSCC tissues and cell lines compared with normal oral keratinocytes. Functional assays demonstrated that its silencing limits the proliferation and invasiveness of cancer cells. Mechanistically, circFOXO3 RNA binds and sequesters miR-214, preventing KDM2A mRNA degradation and leading to its overexpression. Increased KDM2A protein levels promote the proliferation and invasiveness of OSCC cells. This study is the first to demonstrate that KDM2A plays a key role in OSCC progression and invasion, highlighting its potential as a novel therapeutic target for oral cancer [127]. In HNSCC, inactivation of the histone methyltransferase NSD1 leads to loss of H3K36me2 and accumulation of the repressive mark H3K27me3 at the promoters of the chemokines CXCL9 and CXCL10, resulting in T-cell exclusion and poor response to PD-1 blockade. Inhibition of KDM2A restores H3K36me2 levels, reactivates chemokine expression, and promotes T-cell infiltration into NSD1-deficient tumors. Notably, KDM2A suppression reduced tumor growth only in immunocompetent models, highlighting its immunomodulatory role. These findings identify KDM2A as a potential target to overcome immune exclusion and enhance immunotherapy efficacy in HNSCC [56].

### 5.3. KDM3A/KDM3B/KDM3C

The histone demethylases of the KDM3 family—comprising KDM3A, KDM3B, and KDM3C—specifically remove mono- and di-methyl groups from lysine 9 on histone H3 (H3K9me1/2), thereby modulating chromatin accessibility and transcriptional programs. The exact function of the KDM3 family demethylases in HNSCC is not fully understood. However, dysregulated expression of KDM3 has been reported in multiple cancer types, including HNSCC, where they act as an important epigenetic regulator of tumor progression and metastasis.

Overexpression of KDM3A has been correlated with increased expression of its downstream oncogenic target Adrenomedullin (ADM), which promotes cell proliferation and tumorigenesis. High nuclear levels of KDM3A represent an unfavorable prognostic factor, associated with enhanced lymph node metastasis and an over ten-fold increase in metastatic risk in HNSCC patients [128]. On the other hand, in EBV-associated nasopharyngeal carcinoma (NPC), upregulation of miR-155, partly induced by the viral oncoproteins LMP1 and LMP2A, suppresses KDM3A expression, a finding that correlates with advanced lymph node (N) stage and poor prognosis [67]. These findings collectively indicate that KDM3A plays a dual, context-dependent role in HNSCC pathogenesis: its overexpression drives tumor progression by demethylating repressive histone marks, whereas its loss in virally transformed tumors reflects epigenetic reprogramming associated with aggressive disease. Thus, modulation of KDM3A activity may represent a promising therapeutic avenue in specific molecular subtypes of HNSCC.

In contrast, KDM3B and KDM3C have not been functionally characterized in HNSCC and remain candidates for future investigation.

### 5.4. KDM4A/KDM4B/KDM4C/KDM4D

The KDM4 histone demethylase gene family includes six genes—KDM4A-F, of which KDM4E and KDM4F were reported as noncoding pseudogenes. KDM4A-D demethylases play a key role in epigenetic control of gene expression by removing methyl groups from lysine residues on histone H3 (primarily H3K9me2/3 and H3K36me2/3), thereby promoting chromatin relaxation and activating specific transcriptional programs. KDM4A, KDM4B, and KDM4C are broadly expressed in many normal human tissues, whereas KDM4D is present at low levels in normal tissues and is expressed primarily in the testes. KDM4A dysregulation is observed in multiple cancer types, and its overexpression correlates with increased aggressiveness and higher proliferation, promoting migration, metastasis, and treatment resistance in cancer cells. A key role for KDM4A in promoting cancer progression was demonstrated in HNSCC, where it interacts with the transcription factor Lymphoid enhancer-binding factor 1 (LEF1), an important regulator of the Wnt-β-catenin pathway. As a result of this interaction, KDM4A is recruited by LEF1 to the LATS2 promoter, leading to silencing of LATS2 expression, increased cell proliferation, and inhibition of apoptosis. In vivo studies in mouse models have shown that silencing KDM4A expression significantly limits OSCC cells’ ability to initiate and sustain tumor growth, confirming its pro-tumor activity. Functionally, KDM4A acts as a transcriptional co-regulator, conferring a proliferative advantage and resistance to apoptosis of cancer cells [50]. Another publication also reports a significant oncogenic role of KDM4A in NPC, acting through epigenetic activation of the HIF1α/DDIT4/mTOR signaling axis. KDM4A removes the repressive H3K9me3 marks at the HIF1α gene promoter, increasing its expression, which in turn activates DDIT4 and the mTOR pathway. Again, this signaling cascade promotes proliferation, migration, and invasion of cancer cells and inhibits their apoptosis. KDM4A downregulation (via siRNA or the JIB-04 inhibitor) effectively inhibits NPC progression in vitro and in vivo by increasing H3K9me3 levels and HIF1α promoter methylation. In turn, this decreases HIF1α and DDIT4 expression and suppresses mTOR pathway activation. Thus, it has been repeatedly shown across multiple cell types and conditions that KDM4A knockdown and pharmacological inhibition of its enzymatic activity significantly reduce cancer cell proliferation, migration, and invasion, and enhance apoptosis. This repeated, independent functional association across multiple studies confirms that KDM4A is an attractive therapeutic target for NPC and suggests the potential use of JIB-04-type inhibitors [51]. However, JIB-04 is a pan-inhibitor affecting multiple KDMs, making it difficult to attribute the observed effects exclusively to KDM4A inhibition. In contrast, the inhibitor ML324 is a selective inhibitor targeting the KDM4 family, allowing for a more direct link between its biological action and inhibition of this family. Pharmacological inhibition of the KDM4 family with the compound ML324 induces robust apoptosis in HNSCC cells, with relatively minor changes in cell cycle progression, and alters the expression of key genes that regulate cell survival and cycle progression. This confirms the pro-oncogenic role of KDM4 and identifies it as a promising therapeutic target in HNSCC, also in combination with EGFR/PI3K inhibitors [68].

KDM4C was also identified as a critical epigenetic regulator in HNSCC. KDM4C knockdown has significantly reduced cancer cell migration, invasion, and metastasis in both in vitro and in vivo models. A similar effect was observed with the inhibitors myricetin and BPRKD022S0 (22S0). Both are relatively specific KDM4 inhibitors that increased H3K9me3 levels, reduced cell viability, and suppressed heme metabolic pathways. Mechanistically, KDM4C cooperates with transcriptional regulator GATA1 to regulate heme metabolism genes, particularly ferrochelatase (FECH), with FECH overexpression rescuing proliferation defects induced by KDM4C depletion. High KDM4C and GATA1 expression correlate with advanced tumor stage and poor prognosis in HNSCC patients. At the same time, KDM4 inhibitors may be promising therapeutic targets for patients with increased KDM4C expression [129].

KDM4B and KDM4D have not been investigated in HNSCC and remain untested members of the family. In terms of therapeutic relevance and clinical evidence, KDM4A and KDM4C show moderate-strength HNSCC-specific data from functional OSCC and NPC models, as well as patient IHC data. A critical caveat regarding selectivity for these inhibitors is that the pan-KDM inhibitor JIB-04 supports most of the mechanistic data, but one cannot cleanly attribute effects to KDM4 alone; the selective inhibitor ML324 and the natural compound myricetin provide firmer support. The KDM4C-GATA1-FECH heme metabolism axis is an HNSCC-specific vulnerability with no parallel in other cancer types, making it a particularly interesting priority for further characterization and functional or clinical validation. Zavondemstat (TACH101) has entered a first-in-human basket trial (NCT05076552) that includes HNSCC, but no subgroup data is available. Combination treatments with EGFR/PI3K inhibitors are well-motivated and represent the most credible near-term preclinical evidence required before HNSCC-specific clinical testing can be designed.

### 5.5. KDM5A/KDM5B/KDM5C/KDM5D

The KDM5 family of histone demethylases comprises four isoforms: KDM5A, KDM5B, KDM5C, and KDM5D. All isoforms exhibit high sequence homology and catalyze the demethylation of di- and trimethylated H3K4 states (H3K4me2/3), resulting in monomethylated H3K4 (H3K4me1). These enzymes are characterized by the presence of a catalytic JmjC domain, a JmjN domain, an ARID DNA-binding motif, a C5CH2-type zinc finger domain, and PHDs [138,139,140]. The function of enzymes in the KDM5 family is highly context-dependent and varies across tumor types; these demethylases exhibit a dual role, acting as both oncogenes and tumor suppressors. KDM5A, KDM5B, and KDM5C are frequently overexpressed across multiple cancer types, contributing to tumor progression by promoting cell proliferation and migration, ultimately leading to therapy resistance. In contrast, KDM5D expression is often very low, and the demethylase itself acts as a tumor suppressor by regulating signaling pathways, such as p38α MAPK, thereby inhibiting tumor growth and metastasis [61,62,63].

Significant overexpression of KDM5B has been observed in HNSCC, and its high expression correlates positively with tumor cell proliferation (Ki-67). Functional silencing of KDM5B inhibits HNSCC cell growth in vitro and in vivo, induces G1 cell cycle arrest and early apoptosis (including through downregulation of Bcl-2 family proteins. This suggests that KDM5B may function in part as a proliferation-inducing oncogene [52]. KDM5B overexpression has also been observed in subsequent HNSCC studies. Specifically, high levels of KDM5B expression and activity correlate with malignant tumor progression, lymph node metastasis, and recurrence in patients, as well as shorter overall and relapse-free survival (RFS) [130]. Another study showed that KDM5B is significantly overexpressed in human laryngeal squamous cell carcinoma. As in the previous study, high KDM5B levels were associated with advanced disease, metastasis, recurrence, and poorer 5-year survival [131]. Therefore, KDM5B overexpression represents an independent, unfavorable prognostic factor, making it a promising prognostic biomarker in HNSCC. In OSCC, pharmacological inhibition of KDM5B with CPI-455, a selective inhibitor of the KDM5 family, has been shown to effectively suppress some features of stem-like cancer cells (reductions in sphere formation, tumor formation, CD44+ and ALDH-high fractions), but does not abolish all functions of this protein. At the same time, the results reveal an important, demethylase-independent component of JARID1B action that remains insensitive to catalytic inhibition, suggesting that future therapeutic strategies based on JARID1 inhibitors should be considered in combinatorial cancer therapy [70]. It should be noted that EZH2 inhibitors, such as Tazemetostat, and KDM6 inhibitors exert opposite effects on H3K27me3 levels: EZH2 inhibition reduces these repressive marks, whereas KDM6 inhibition preserves or increases them. Accordingly, these approaches are relevant in different therapeutic contexts rather than being redundant. It should be noted that KDM5D is not uniformly a tumor suppressor in HNSCC: one HNSCC-specific study identified elevated KDM5D expression as a functional contributor to platinum-resistant cells. In these cells, disruption of the KDM5D-AURKB axis exposed a collateral vulnerability to inducing mitotic catastrophe. Accordingly, cisplatin combined with AURKB inhibitors strongly suppressed tumor growth in vivo. Nevertheless, this evidence is from a single study and may be sex-restricted because KDM5D is Y-linked [65]. KDM5B, in contrast, is reported as a potential oncogenic driver that promotes EMT and metastasis via activating Wnt/β-catenin signaling [141].

### 5.6. KDM6A/KDM6B/KDM6C

The KDM6 family comprises three paralogues: KDM6A (UTX), KDM6B (JMJD3), and KDM6C (UTY), discovered in 2007. KDM6A and KDM6B possess robust H3K27me2/me3 demethylase activity, as confirmed by in vitro kinetics and numerous functional studies [142]. In contrast, KDM6C retains residual, male-specific H3K27me2/me3 demethylase activity [143] but acts primarily as a scaffold for chromatin complex recruitment through protein–protein interactions. Its cancer-specific functions remain incompletely characterized.

In HNSCC, pharmacological inhibition of LSD1 and KDM6B with TCP and GSK-J1 leads to strong inhibition of proliferation, induction of apoptosis and senescence in HNSCC cells in vitro, and reduced tumor growth and progression in vivo, accompanied by regulation of cell-cycle-related genes. Other studies conducted in the HNSCC model revealed that inhibiting KDM6A and KDM6B activity using the GSK-J1 inhibitor or by silencing these genes with siRNA increases H3K27me3 levels, leading to chromatin condensation, transcriptional repression, and increased sensitivity of tumor cells to radiotherapy. High-level KDM6A expression is associated with a favorable prognosis and improved locoregional control after radiotherapy, whereas KDM6B overexpression correlates with poorer OS, suggesting distinct prognostic implications for both enzymes in HNSCC [48]. However, another study indicated that KDM6A plays an oncogenic rather than a suppressor role in OSCC. KDM6A overexpression correlates with poorer overall and relapse-free survival (RFS) in patients after tumor resection. Furthermore, pharmacological inhibition of KDM6A with the drug GSK-J4 in OSCC cell lines reduces migration, invasion, cell-cycle progression, and EMT in a dose-dependent manner, supporting KDM6A as a potential therapeutic target [45]. Together, these findings suggest that KDM6A’s prognostic impact in HNSCC is highly subsite-specific: it may be favorable in mixed HNSCC cohorts where radiotherapy response dominates outcomes [48], but unfavorable in OTSCC [45]. Prospective, stratified analyses that distinguish subsites (e.g., oral cavity), HPV status, and treatment modality are required before KDM6A can be deployed as either a prognostic biomarker or a therapeutic target.

In esophageal squamous cell carcinoma (ESCC), KDM6B overexpression correlates with lymph node metastasis, whereas KDM6B silencing limits cancer cell proliferation and metastasis in vitro and in vivo. KDM6B, through its H3K27 demethylase activity, increases C/EBPβ transcription and enhances TNFα/NFκB signaling, while pharmacological inhibition with GSK-J4 inhibits cancer cell growth [132]. When it comes to their therapeutic relevance and evidence level, KDM6A and KDM6B present the most complex prognostic picture, with subsite-specific and context-dependent effects that resist simple characterization. The most consistent and clinically relevant finding is the apparent radiosensitization: inhibition of KDM6 activity with the inhibitor GSK-J1/J4 increases H3K27me3 methylation marks, results in locally condensed chromatin, and improves radiotherapy response in HNSCC models. These findings suggest KDM6A and KDM6B as the strongest candidates and the most promising rationale for combinatorial use. Dual LSD1/JMJD3 inhibition (using tranylcypromine TCP and GSK-J1) is simultaneously synergistic in HNSCC and represents a conceptually important proof of principle for two-KDM targeting. A critical limitation is that GSK-J1/J4 are only experimental compounds, not yet in any active clinical development. This relatively strong evidence, nevertheless, suggests that the field may benefit from more selective, clinical-grade KDM6 inhibitors and that HNSCC-specific trials may become feasible. Future studies should stratify patients by tumor localization or subsite (e.g., oral cavity vs. larynx/pharynx), HPV status, and prior radiotherapy; they should also investigate baseline H3K27me3 levels to resolve the currently conflicting prognostic data. This line of evidence, however, should be able to identify the patient subset most likely to benefit from such therapies.

### 5.7. KDM7A/KDM7B/KDM7C

As with the KDM6 family, the roles of KDM7 family members in cancer vary depending on the context. In some models, they behave as tumor suppressors. In contrast, in other models, these epigenetic enzymes may rather enhance pro-tumor programs related to proliferation, EMT, migration, plasticity, and CSC characteristics. In practice, KDM7A and KDM7B more often exhibit a function similar to that of “epigenetic oncogenes”, whereas KDM7C resembles that of a tumor suppressor gene. Once again, this target-specific difference underscores the need to always consider their roles in the specific context of the tumor type [133], subtype, and localization. Research on KDM7B suggests it likely plays an oncogenic role: its overexpression in tumor tissues correlates with poor prognosis, and elevated KDM7B levels promote proliferation, invasion, and metastasis. In HNSCC, overexpression of KDM7B in laryngeal and hypopharyngeal cancers indicates that this demethylase plays a pro-oncogenic role, as it is associated with higher tumor stage, higher risk of recurrence, and shorter overall and recurrence-free survival. KDM7B promotes HNSCC progression by regulating H3K9me2 and H3K27me2 levels, thereby reinforcing its importance as an unfavorable prognostic marker and a potential therapeutic target in HNSCC [46]. The KDM7 family is slowly emerging as a promising yet still poorly explored therapeutic target in HNSCC. To date, there are no dedicated studies of KDM7 inhibitors in HNSCC. Still, the development of selective compounds targeting KDM7A/B and their efficacy in other tumor models would represent a solid rationale for preclinical testing in HNSCC cell lines and models, particularly in the context of resistance to radiotherapy, chemotherapy, and targeted therapy.

### 5.8. KDM8

KDM8 (JMJD5) is a histone demethylase that specifically demethylates H3K36me2. However, more recent studies indicate that it does not actually play a primary role as a histone lysine demethylase but rather acts primarily as an arginine oxyhydroxylase [144,145].

KDM8 overexpression in OSCC promotes tumor cell migration, invasion, and survival, thereby making KDM8 a potentially unfavorable prognostic marker in HNSCC. KDM8 silencing leads to tumor growth inhibition in vitro and in vivo by reversing EMT (↑E-cadherin, ↓N-cadherin/vimentin), inducing p53-dependent apoptosis, and decreasing nuclear NF-κB activity, providing strong support that KDM8 is an attractive therapeutic target and that its pharmacological inhibition may represent a novel treatment strategy for HNSCC [47]. Another study also demonstrated a correlation between high KDM8 overexpression in OSCC and larger tumor size, the presence of cervical metastases, and poorer survival, confirming its importance as an unfavorable prognostic marker and marker of metastasis in HNSCC. Additionally, the study identifies the MTA1-KDM8 axis as a significant regulator of OSCC progression. It indicates that the inhibitor Silibinin, by reducing the expression of both MTA1 and KDM8, may inhibit tumor cell growth in tongue cancer cell lines and PDX models for oral cancer. Silibinin may be a promising drug targeting specifically this axis in HNSCC therapy [134].

In summary, at present, the clinical translation of KMT and KDM inhibitors in cancer, especially in HNSCC, is in its very early stages. Future trials should rely on informative biomarkers to identify suitable patients. At the very minimum, future trials should carefully establish the HPV/p16 status for all patients who are enrolled and avoid heavily pretreated patients. In addition, the functional expression and activity of the corresponding drug targets should be evaluated prospectively. In particular, for compounds such as Tazemetostat targeting EZH2, it would be critical to assess not only EZH2 expression and activity but also H3K27me3 levels and the expression of putative target genes. In addition, the opposing status of NSD1 activity should be evaluated in the tumor tissue of such patients. In addition, for drugs targeting candidates like LSD1, expression and activity of this enzyme would need to be assessed, ideally combined with evaluating the presence of stemness-related features in the patient’s cancer tissue.

## 6. HPV Status as a Determinant of Epigenetic Vulnerability in HNSCC

HPV status is the single most important biological variable for interpreting KMT/KDM biology in HNSCC, yet it has been insufficiently integrated into the epigenetic “landscape” mirrored in the literature. HPV-positive and HPV-negative HNSCC are not merely prognostically distinct. These represent distinct tumor subtypes that differ significantly in mutational landscape, chromatin architecture, immune microenvironment, and operative epigenetic (and genetic) dependencies. Mixing up or not differentiating the two groups severely obscures the therapeutic options. However, this conflation remains a major contributor to inconsistencies in published enzyme expression and functional data.

In HPV-negative HNSCC, the dominant epigenetic alterations discussed above (including NSD1 loss, SMYD3 overexpression, EZH2 overexpression, and G9a/EHMT2 activity) are most consistently described and best mechanistically supported. NSD1 loss defines a distinct chromatin subtype characterized by depletion of H3K36me2 marks, accumulation of compensatory H3K27me3 marks, an immune-cold TME, and generally higher cisplatin sensitivity [75]. SMYD3 is overexpressed in approximately 80% of HPV-negative tumors. Its depletion may convert the TME of HNSCC lesions from cold to hot immune status [54,55]. Similarly, high EZH2 expression and its correlation with advanced stage and poor prognosis have also been established predominantly in HPV-negative cohorts [83] but are not observed in HPV+ tumors. These observations collectively suggest that the most clinically actionable KMT targets in HNSCC are, at present, largely disease-specific (or more so for HPV-negative disease).

In HPV-positive HNSCC, the epigenetic landscape is fundamentally different. The HPV E6/E7 oncoproteins themselves drive chromatin reprogramming: E6/E7 upregulate KDM2B through c-MYC overexpression and miR-146a-5p suppression, linking viral oncoproteins directly to a KDM that promotes proliferation and suppresses senescence [126]. More recently, HPV E6/E7 were shown to induce NSD2, thereby increasing activating H3K36me2 marks at selected loci and driving proliferation and inhibiting epithelial differentiation in HPV-positive models [146]. This creates an almost mirror image of the logic observed in HPV-negative disease: whereas HPV-negative tumors are often shaped by NSD1 loss and H3K36 deficiency, HPV-positive tumors may instead exploit and benefit from NSD2-driven H3K36me2 methylation. This helps them to maintain an undifferentiated, highly proliferative state. Low expression of NSD2 (and NSD1/NSD3) in HPV-positive HNSCC correlates with significantly worse overall survival. Again, this is the opposite of the relationship observed in HPV-negative disease [60]. These opposing associations may indicate that the NSD-family expression has fundamentally different prognostic meaning across the two subtypes. In contrast, the demethylase KDM2B, whose overexpression is enriched in HPV-positive tumors, may represent a more specific therapeutic vulnerability in this subgroup and may be better suited for this tumor subtype than the methyltransferase targets currently dominating the field.

The striking context-dependence also extends beyond simple HPV stratification. The function of KMT2D exemplifies this: it is lost in laryngeal and hypopharyngeal tumors where it suppresses proliferation, but it is overexpressed and likely acts pro-tumorigenic in OSCC and HPV-positive OPSCC. In these tumors, it maintains stemness and Wnt signaling [57,58,59]. KDM6A is another example of differential, context-dependent functions: it carries favorable prognostic significance in radiotherapy-dominated mixed HNSCC cohorts but shows adverse significance in oral-tongue SCC [45,48]. These few examples argue strongly that future studies and trials should be stratified by HPV/p16 status, anatomical subsite/tumor localization, and prior treatment. These are minimum requirements, not just optional refinements. HPV status and tumor subsite are not secondary nuances or side notes; they must be seriously considered and acknowledged for patient stratification. They also represent primary biological variables that determine which epigenetic dependency and “connectivity” is truly operative in each patient.

## 7. KMT/KDM Inhibitors in the Broader Epigenetic Landscape: Complementarity with HDAC and DNMT Inhibitors

A question naturally arising from this review is why KMT/KDM inhibitors deserve attention alongside—or instead of—the more clinically mature HDAC and DNMT inhibitor classes. The answer is not that KMT/KDM inhibitors are globally superior. HDAC inhibitors (e.g., Vorinostat, Romidepsin, and Entinostat) and DNMT inhibitors (e.g., Azacitidine and Decitabine) have a considerably longer clinical track record, broader regulatory approvals, and well-characterized toxicity profiles. They act broadly on chromatin accessibility and DNA methylation respectively, and several of these compounds have demonstrated activity in hematologic malignancies with ongoing solid-tumor investigation.

The potential added value of KMT/KDM targeting lies in their relative pathway specificity and biomarker tractability rather than in broad epigenetic potency. Where a defined genetic alteration in tumors exists, such as NSD1 loss, EZH2 overexpression, SMYD3-driven immune exclusion, or G9a-mediated EMT/CSC—it creates a dependency on a specific enzyme. In these cases, a targeted inhibitor can theoretically exploit that dependency as a specific vulnerability of the tumor. Ideally, it can then act with less off-target chromatin disruption than the usually very broad HDAC or DNMT inhibitors. Conversely, when the goal is to broadly lower the epigenetic barrier of tumor cells to immune recognition or differentiation, for example, in immunologically uncharacterized HNSCC, a broad-acting DNMT inhibitor may be more reliable across a larger and more heterogeneous patient population. The most promising near-term strategy is therefore likely to identify the most promising combinatorial therapies on an individualized basis. For example, a DNMT or HDAC inhibitor may be used to generally lower the chromatin barrier. This can then be combined with a KMT/KDM inhibitor to more specifically exploit a vulnerability defined by the patient’s individual genotype and with a checkpoint inhibitor to capitalize on the restored immune signal. None of these combinations have been formally tested in adequately designed HNSCC trials, but many of these suggestions may represent future directions for the next phase of clinical development that await testing.

## 8. Rational Combination Strategies: Organizing KMT/KDM Inhibitors by Treatment Modality

The preceding sections have established that KMT/KDM inhibitors are more likely to act as combination sensitizers than as standalone cytotoxic agents. Organizing this rationale by treatment modality—rather than by enzyme—makes the translational logic clearer and maps more directly onto how HNSCC is treated clinically.

### 8.1. Combinations with Radiotherapy

Radiotherapy is the backbone of curative-intent treatment for locally advanced HNSCC, making radio-sensitization the most immediately clinically relevant application of KMT/KDM inhibitors. The strongest preclinical rationale here involves KDM6A/KDM6B and G9a/EHMT2. KDM6 inhibition with GSK-J1/J4 increases repressive H3K27me3, condenses chromatin, and reduces transcriptional recovery, thereby limiting recovery and increasing tumor-cell death after radiation-induced damage; this effect is well replicated across HNSCC models and has a clear mechanistic explanation [48]. G9a loss promotes TMEM27-dependent ferroptosis after irradiation, markedly enhancing radiosensitivity in vitro and in xenograft models [49]. Both represent candidate pretreatment radiosensitization strategies: administered before or concurrently with radiotherapy to lower the chromatin-mediated recovery threshold. Critically, neither GSK-J1/J4 nor available G9a inhibitors are yet in clinical-stage development, meaning the immediate priority is advancing a clinical-grade KDM6 or G9a inhibitor to enable combination testing. The rationale for sequencing these agents before radiotherapy, rather than during or after it, is supported by biology: chromatin compaction should precede, not follow, the radiation insult.

### 8.2. Combinations with Platinum-Based Chemotherapy

Cisplatin resistance is the predominant mechanism of chemotherapy failure in HNSCC. Two mechanistically distinct epigenetic routes to platinum sensitization have emerged from the reviewed evidence. First, G9a/EHMT2 inhibition directly lowers the glutathione-mediated detoxification barrier: G9a transcriptionally activates GCLC, upregulating glutathione synthesis; its inhibition reduces this buffer and restores cisplatin sensitivity in resistant cell lines. This is a direct, on-mechanism sensitization strategy that would be most rational in G9a-high, cisplatin-resistant tumors identified by H3K9me2 readout and GCLC expression. Second, NSD1-deficient tumors exhibit intrinsic cisplatin sensitivity due to altered H3K36me2/H3K27me3 balance and compromised DNA repair programs; this NSD1-loss vulnerability should be exploited by selecting NSD1-mutant patients for cisplatin-containing regimens in prospective trials. LSD1 inhibition also increases 5-FU sensitivity in OSCC models [66,117], providing a third route—though this data rests on cell lines and requires validation in patient-derived models before clinical extrapolation.

### 8.3. Combinations with EGFR/PI3K-Targeted Therapy

KDM4A interacts with LEF1 to silence LATS2 and activate Wnt/β-catenin signaling, and KDM4A/C upregulate the HIF1α/DDIT4/mTOR axis—pathways that directly converge with EGFR and PI3K signaling networks [50,51]. The selective KDM4 inhibitor ML324 synergizes with EGFR/PI3K inhibitors in HNSCC cell models [68], providing the strongest mechanistic argument in this review for a KDM/targeted-therapy combination. The rationale is that KDM4 inhibition collapses a chromatin-mediated bypass route that allows tumor cells to survive EGFR/PI3K blockade; in this model, the epigenetic agent acts as an adaptive-resistance suppressor rather than a direct cytotoxin. Translational priority: KDM4A/C expression and EGFR pathway status should be co-assessed in future biomarker studies to define the patient subset most likely to benefit from this combination.

### 8.4. Combinations with Immune Checkpoint Blockade

The most compelling combination rationale—and the one with the broadest evidence base—is KMT/KDM inhibition combined with immune checkpoint blockade. As established in Section 3.1, at least four enzymes (EZH2, SMYD3, LSD1, and NSD1-loss/KDM2A axis) independently suppress the MHC-I/IFN/CXCL9-10 immune axis. This convergence predicts that several mechanistically different KMT/KDM inhibitors could serve as immune-sensitizing agents, before or alongside anti-PD-1/PD-L1 or ICI therapy. The strongest individual evidence exists for SMYD3: depletion with antisense oligonucleotides in syngeneic HPV-negative HNSCC (animal) models achieved 50% complete cures in combination with anti-PD-1. They showed largely restored CD8+ T-cell infiltration and de-repressed antigen-presentation machinery [54,55]. As outlined above, the combination of EZH2 inhibition with ICIs has been assessed clinically (Tazemetostat and Pembrolizumab, NCT04624113). Unfortunately, this small Phase I trial was conducted in an unselected cohort of refractory patients without biomarker stratification. This trial produced no objective responses [72]. We have also discussed that LSD1 inhibition raises PD-L1, providing a strong mechanistic rationale for combination with anti-PD-1 blockade [42]. The NSD1-deficient/KDM2A axis links a genotype-defined chromatin state to T-cell exclusion. This may be reversible by KDM2A inhibition in immunocompetent models [56]. Taken together, the immune combination is the direction that appears most likely to generate the first convincing HNSCC clinical results, but this may only be successful if future trials are designed with acknowledgment of the patients’ HPV status, target gene expression, immune status profiling, and include assessment of histone marks as part of the pharmacodynamics and as surrogate markers. These are all potential lessons from the disappointing Tazemetostat experience.

## 9. Conclusions

A growing body of experimental data in recent years indicates that both KMTs and KDMs play key roles in shaping treatment resistance and the aggressive phenotype of HNSCC. However, their contribution is more heterogeneous than initially appreciated: EZH2, SMYD3, LSD1, G9a/EHMT2, KDM4A/C, KDM5B, KDM7B, and KDM8 act as oncogenic dependencies, whose overexpression or increased activity promotes epigenetic silencing of tumor suppressors. These genes sustain stemness and can also drive therapy resistance. In contrast, NSD1 and KMT2C/D are frequently lost in specific HNSCC subtypes and define subtype-specific vulnerabilities. NSD1-deficient tumors accumulate compensatory H3K27me3 marks and may become sensitive to EZH2 inhibition. KDM5D and KDM6A most frequently act as tumor suppressors. This outstanding level of heterogeneity indicates that therapeutic targeting of methylation-related key players in patients should be biomarker-driven and cannot be applied as a one-size-fits-all, generalized strategy.

A more detailed characterization of the roles of individual KMTs and KDMs involved in cancer initiation, progression, metastasis, and treatment resistance is therefore crucial for the rational design of personalized therapies in HNSCC. Nevertheless, the use of broad-spectrum agents, as well as more selective KMT/KDM inhibitors, represents a potentially rational strategy to overcome drug and therapy resistance. This is ideally addressed in combination with standard chemotherapy and radiotherapy, and, in HNSCC, with EGFR-targeted therapy and immunotherapy, which may improve treatment efficacy. This growing body of experimental and functional evidence—mostly in cancer models—justifies further mechanistic preclinical studies, and translational studies in patients and clinical trials [147]. However, several limitations of the existing body of evidence must be acknowledged: many studies rely on small patient cohorts or heterogeneous HNSCC datasets, HPV status is frequently not accounted for as a confounding variable, TCGA data are routinely re-analyzed without independent validation, and longitudinal data tracking epigenetic changes during treatment or at the time of relapse remain scarce.

Preclinical data support histone methylation as a relevant HNSCC-specific vulnerability, but clinical translation remains very limited. The most mature evidence concerns EZH2, yet the only published HNSCC trial (tazemetostat plus pembrolizumab) showed only modest activity in an unselected cohort and was discontinued, and the PANTHERAS valemetostat trial was withdrawn before efficacy data; no EZH1/2 program in HNSCC can currently be considered established. As detailed in Section 3.3 and Section 3.4, future studies should prioritise biomarker-enriched designs—notably EZH2-high or NSD1-altered tumors—combined with standard-of-care and include mandatory on-treatment biopsies documenting target engagement (e.g., H3K27me3, H3K9me2, H3K4me2) and immune-microenvironment changes.

## Figures and Tables

**Table 2:** HNSCC-specific clinical trials of KMT/KDM inhibitors. Only trials with a registered HNSCC-specific arm or completed HNSCC cohort are included. Broader KMT/KDM inhibitor landscape across tumor types is summarized in Supplementary Table S1.

Key Reference/NCT Number	HNSCC Preclinical Evidence	HNSCC Clinical Data	Approved/ Main Clinical Indications	Regulatory Approval	Highest Clinical Stage	Inhibition Mechanism	Gene Target (KMT/KDM)	Drug Name (INN/Code/Brand)
NCT05879484	Dual EZH1/2 inhibition may overcome EZH1 compensatory activation and represent a second-generation PRC2-targeting strategy for HNSCC.	NCT05879484 (PANTHERAS): active trial in HPV-negative RM-HNSCC and sinonasal SCC in combination with pembrolizumab.	ATLL (Japan); solid tumors; active HNSCC program	Approved in Japan (2023) for ATLL	Phase 1b/2	SAM-competitive dual EZH1/2 inhibitor; oral once-daily	EZH1; EZH2	Valemetostat (DS-3201b/Ezharmia^®^)
NCT04624113; [72]	EZH2 is overexpressed in HNSCC; inhibition of EZH2 may restore antitumor immunity and improve responses to anti-PD-1 in preclinical models.	NCT04624113: Phase 1 trial in RM-HNSCC + pembrolizumab showed no objective responses (5/12 SD); phase 2 not opened; all trials halted.	Previously: follicular lymphoma, epithelioid sarcoma	Withdrawn globally (March 2026)	Phase 1/2/3 (halted)	SAM-competitive orthosteric SET-domain inhibitor; oral	EZH2	Tazemetostat (EPZ-6438/Tazverik^®^)
NCT02875223; [97]	High-throughput screening in HNSCC identified pulrodemstat as a potent KDM inhibitor that induces apoptosis and reduces proliferation; KDM1A overexpression correlates with malignancy in HNSCC.	No registered HNSCC clinical trial.	NEN, solid tumors	Not approved	Phase 1/2	Reversible non-covalent LSD1 inhibitor; oral	KDM1A	Pulrodemstat (CC-90011)
NCT02912182; [98]	LSD1 is overexpressed in HNSCC and promotes EMT and CSC phenotypes; preclinical data support combining LSD1 inhibition with immune checkpoint blockade and radiotherapy.	No registered HNSCC clinical trial; no published HNSCC-specific clinical results.	AML, SCLC	Not approved	Phase 1/2	TCP-derived covalent LSD1 inhibitor; oral once-daily	KDM1A	Iadademstat (ORY-1001/iadademstat)
NCT05076552; [99]	KDM4A/C overexpression has been reported in OSCC/HNSCC; KDM4 inhibition induces apoptosis and sensitizes HNSCC cells to EGFR/PI3K inhibitors.	NCT05076552: first-in-human basket trial; no HNSCC-specific subgroup results reported.	Advanced solid tumors; HNSCC eligible in basket trial	Not approved	Phase 1	Alpha-ketoglutarate, a competitive JmjC active-site inhibitor; oral	KDM4A; KDM4B; KDM4C; KDM4D	Zavondemstat (TACH101/QC8222)
[71]	In HNSCC, dual inhibition of KDM6B and LSD1 (GSK-J1/J4 + tranylcypromine) shows synergistic antitumor effects in vitro and in vivo; KDM6 inhibition may radiosensitize HNSCC.	No HNSCC clinical data.	Research use only	Not approved (tool compound)	Preclinical only	JmjC active-site inhibitor; cell-permeable prodrug tool compound	KDM6A; KDM6B	GSK-J4 (tool compound)
[68]	Selective KDM4 inhibitor that induces apoptosis and reduces growth of HNSCC cell lines (CAL27, FaDu); enhances efficacy of EGFR/PI3K inhibitors.	No HNSCC clinical data.	Research use only	Not approved (tool compound)	Preclinical only	JmjC active-site competitive KDM4-selective inhibitor	KDM4A-D	ML324 (tool compound)
[50,51]	Pan-KDM inhibitor with activity in nasopharyngeal carcinoma and HNSCC models; reduces proliferation, alters HIF-1alpha signaling, and may synergize with chemoradiotherapy.	No HNSCC clinical data.	Research use only	Not approved	Preclinical only	JmjC competitive pan-KDM tool compound; not clinically developed	Multiple KDMs	JIB-04 (pan-KDM tool)

## Data Availability

No new data were created or analyzed in this study. Data sharing is not applicable to this article.

## Supplementary Materials

The following supporting information can be downloaded at: https://www.mdpi.com/article/10.3390/cancers18132170/s1, Table S1: Broader landscape of KMT and KDM inhibitors across HNSCC.

## Abbreviations

The following abbreviations are used in this manuscript:

2-OG	α-ketoglutarate
5-FU	5-fluoruracil
ADM	adrenomedullin
APM	antigen-processing and presentation machinery
ARID	AT rich interaction domain
ASOs	antisense oligonucleotides
BHC/BRAF	histone deacetylase complex
CDK	cyclin-dependent kinase
CNV	copy-number amplification
CSC	cancer stem cell
EBV	Epstein–Barr virus
EGFR	epidermal growth factor receptor
EMT	epithelial–mesenchymal transition
ESCC	esophageal squamous cell carcinoma
FECH	ferrochelatase
H3K4me1	monomethylated lysines
H3K4me1/2	demethylation of histone H3 lysines
H3K4me3	trimethylation of histone H3 lysine 4
H3K36me2	dimethylation of lysine 36 on histone H3
H3K79	methylation of histone H3 at position K79
HCC	hepatocellular carcinoma
HNC	head and neck cancers
HNSCC	head and neck squamous cell carcinoma
HPV	human papillomavirus
ICI	immune checkpoint inhibitor
ISG	interferon-stimulated gene
JmjC-KDMs	Jumonji C-type demethylase
KDM	histone lysine demethylase
KO	knockout
Kme3	trimethyl marks lysine
KMT	histone lysine methyltransferase
LEF1	Lymphoid enhancer-binding factor 1
LSD1/KDM1A	Lysine-specific demethylase 1
LSD2/KDM1B	FAD-dependent demethylase
MORN	membrane occupation and recognition nexus repeat domain
MYND	myeloid, Nervy and DEAF 1 zinc-finger domain
NPC	nasopharyngeal carcinoma
OPSCC	oropharyngeal squamous cell carcinoma
OS	median overall survival
OSCC	oral squamous cell carcinoma
PD	1 death receptor-1
PFS	progression-free survival
PHD	plant homeodomain
PTM	post-translational modification
REST	RE1-Silencing Transcription Factor
RFS	relapse-free survival
SAH	S-adenosylhomocysteine
SAM	S-adenosylmethionine
SET domain	Su(var)3-9, Enhancer-of-zeste
SMYD3	SET and MYND domain-containing 3
SN2	second-order nucleophilic substitution
TME	tumor microenvironment
TPR	tetratricopeptide repeat domain
