# Supplementary material for: Overcoming Therapeutic Resistance in Head and Neck Squamous Cell Carcinoma (HNSCC): The Role of Histone Methyltransferase and Demethylase Inhibitors
Source: Cancers (Basel). 2026 Jul 6;18(13):2170. doi: 10.3390/cancers18132170 (PMC13359738; doi:10.3390/cancers18132170)
Supplement: Supplementary file 1 [file cancers-18-02170-s001.zip › cancers-4327541-supplementary.pdf]

Supplementary Table S1. Broader landscape of KMT and KDM inhibitors across HNSCC.

Drug	Result / key interpretation	Phase / status	Biomarker / selection	Combination	HNSCC population	Trial ID	Target
Tazemetostat (Tazverik®)	No objective responses; 5/12 patients with stable disease. No EZH2 expression or H3K27me3 selection. Majority had prior anti-PD-1 exposure. Tazemetostat subsequently withdrawn globally due to secondary haematologic malignancies in SYMPHONY-1 (follicular lymphoma). Phase 2 never opened [61].	Phase 1 / completed; drug withdrawn (March 2026)	None (unselected)	Pembrolizumab	R/M HNSCC; prior anti-PD-1 permitted	NCT04624113	**EZH2**
Valemetostat (DS-3201b / Ezharmia®)	Better designed than NCT04624113 - HPV/PD-L1 selection incorporated. Trial listed as withdrawn before efficacy data were available. No published HNSCC results. Dual EZH1/2 inhibition rationale remains biologically sound for NSD1-deficient or EZH2-high HPV-negative disease; would benefit from NSD1 and H3K27me3 co-stratification in future iterations.	Phase 1b/2 / withdrawn (no efficacy data)	PD-L1 CPS; HPV/p16 status	Pembrolizumab	PD-L1-positive, HPV-negative R/M HNSCC; sinonasal SCC	NCT05879484 (PANTHERAS)	**EZH1/2**
Zavondemstat (TACH101)	No HNSCC-specific subgroup results reported. Basket design allows HNSCC enrolment but does not constitute HNSCC validation. Preclinical rationale (KDM4A/C overexpression in OSCC, EGFR/PI3K sensitisation) is solid but unconfirmed clinically. Should be interpreted as early clinical exploration only [64].	Phase 1 / first-in-human; ongoing	None specified	Monotherapy (dose-escalation)	Advanced solid tumors (HNSCC eligible in basket design)	NCT05076552	**KDM4 A-D**
